# Loss of the Thioredoxin Reductase Trr1 Suppresses the Genomic Instability of Peroxiredoxin *tsa1* Mutants

**DOI:** 10.1371/journal.pone.0108123

**Published:** 2014-09-23

**Authors:** Sandrine Ragu, Michèle Dardalhon, Sushma Sharma, Ismail Iraqui, Géraldine Buhagiar-Labarchède, Virginie Grondin, Guy Kienda, Laurence Vernis, Roland Chanet, Richard D. Kolodner, Meng-Er Huang, Gérard Faye

**Affiliations:** 1 Centre National de la Recherche Scientifique, UMR3348, Orsay, France; 2 Institut Curie, Centre de Recherche, Orsay, France; 3 Department of Medical Biochemistry and Biophysics, Umea University, Umea, Sweden; 4 Ludwig Institute for Cancer Research, University of California School of Medicine San Diego, La Jolla, California, United States of America; Instituto de Biociencias - Universidade de São Paulo, Brazil

## Abstract

The absence of Tsa1, a key peroxiredoxin that scavenges H_2_O_2_ in *Saccharomyces cerevisiae*, causes the accumulation of a broad spectrum of mutations. Deletion of *TSA1* also causes synthetic lethality in combination with mutations in *RAD51* or several key genes involved in DNA double-strand break repair. In the present study, we propose that the accumulation of reactive oxygen species (ROS) is the primary cause of genome instability of *tsa1*Δ cells. In searching for spontaneous suppressors of synthetic lethality of *tsa1*Δ *rad51*Δ double mutants, we identified that the loss of thioredoxin reductase Trr1 rescues their viability. The *trr1*Δ mutant displayed a Can^R^ mutation rate 5-fold lower than wild-type cells. Additional deletion of *TRR1* in *tsa1*Δ mutant reduced substantially the Can^R^ mutation rate of *tsa1*Δ strain (33-fold), and to a lesser extent, of *rad51*Δ strain (4-fold). Loss of Trr1 induced Yap1 nuclear accumulation and over-expression of a set of Yap1-regulated oxido-reductases with antioxidant properties that ultimately re-equilibrate intracellular redox environment, reducing substantially ROS-associated DNA damages. This *trr1*Δ -induced effect was largely thioredoxin-dependent, probably mediated by oxidized forms of thioredoxins, the primary substrates of Trr1. Thioredoxin Trx1 and Trx2 were constitutively and strongly oxidized in the absence of Trr1. In *trx1*Δ *trx2*Δ cells, Yap1 was only moderately activated; consistently, the *trx1*Δ *trx2*Δ double deletion failed to efficiently rescue the viability of *tsa1*Δ *rad51*Δ. Finally, we showed that modulation of the dNTP pool size also influences the formation of spontaneous mutation in *trr1*Δ and *trx1*Δ *trx2*Δ strains. We present a tentative model that helps to estimate the respective impact of ROS level and dNTP concentration in the generation of spontaneous mutations.

## Introduction

Reactive oxygen species (ROS) are formed in all oxygen-consuming organisms. ROS can attack almost all cell components and can induce many types of DNA damage that can cause mutations and genome rearrangements. Yeasts like *Saccharomyces cerevisiae*, and other aerobic organisms, have acquired a wide array of mechanisms, including pathways that repair the ROS-induced DNA damage, to prevent the deleterious effects of ROS [1]. Because redox communication occurs between the different cellular compartments [2], the cytosol accumulates endogenous oxidizing compounds arising as byproducts of mitochondrial, peroxisomal and endoplasmic reticulum metabolism. However, under normal growth conditions, steady state levels of ROS in the *S. cerevisiae* cytoplasm are low [3]. Two main redox systems are involved in reducing the level of ROS, the glutathione (GSH) and thioredoxin (Trx) pathways. The GSH pathway involving glutaredoxins is thought to provide a redox buffering function and GSH is the reductant of the glutaredoxins. The thioredoxin redox system comprising the thioredoxins (Trxs) and thioredoxin reductase (Trr) plays a major role in H_2_O_2_ metabolism through the peroxiredoxins [4]. The Trxs are the preferred reductants of the ribonucleotide reductase (RNR) [5] and the 3’-phosphoadenylsulfate reductase (PAPS reductase) [6]. When *S. cerevisiae* cells experience endogenous or exogenous stresses that disturb redox homeostasis, they respond by altering their transcriptional program [7]. Two transcription factors are mainly involved: Yap1 and Skn7, which function in part cooperatively in the peroxide response [8]. Yap1 is the main regulator that controls the expression of *S. cerevisiae* genes encoding most antioxidants, components of glutathione and carbohydrate metabolism, and components of different metal and drug response pathways [8]–[10].

*S. cerevisiae* possesses five peroxiredoxins, which have different sub-cellular localizations. Among them, Tsa1 has the most potent ability to scavenge H_2_O_2_ [11]. In addition to its role in peroxide reduction, Tsa1 is also known to have chaperone activity [12]. Tsa1 is the only peroxiredoxin that causes an elevated Can^R^ mutation rate when individually removed, indicating that Tsa1 is the most important peroxiredoxin for preventing ROS-induced mutations [13]. DNA lesions resulting from oxygen metabolism can lead to formation of mutations by action of replicative DNA polymerases and translesion DNA polymerases. ROS-induced DNA damage also activates checkpoint pathways which could stimulate dNTP production [14]. This up-regulation of dNTP synthesis facilitates the repair of DNA lesions but is associated with higher mutation rates resulting in part from more efficient translesion DNA synthesis [15]. Tang and collaborators [16] have shown that the accumulation of ROS in *tsa1*Δ cells accumulate DNA lesions which, by presumably activating the DNA damage checkpoint, stimulate dNTP production. However, it is not understood whether it is the accumulations of ROS and consequently accumulations of mutagenic DNA lesions or the overproduction of dNTPs that is the primary cause of the elevated Can^R^ mutation rate in *tsa1* mutants.

Homologous recombination is involved in repair of many types of DNA lesions. We have previously shown that combining a *tsa1*Δ mutation with a *rad51*Δ mutation or mutations inactivating other key genes that function in DNA double-strand break (DSB) repair results in cell death [17]. Oxygen metabolism likely takes part in the inviability of *tsa1*Δ *rad51*Δ double mutants since anaerobic conditions were found to restore the viability of *tsa1*Δ *rad51*Δ double mutants [18]. One explanation for this is that in cells growing in aerobic conditions, the absence of Tsa1 results in a high level of DNA damage that is lethal in the absence of key DNA repair pathways such as DSB repair. To better understand the cause of the elevated mutation rate of the *tsa1*Δ mutants and the synthetic lethality of *tsa1*Δ *rad51*Δ double mutants, we screened for spontaneous mutations that suppress synthetic lethality of *tsa1*Δ *rad51*Δ double mutants. We found that mutations in *TRR1*, which encodes the cytosolic thioredoxin reductase, are able to rescue the growth of *tsa1*Δ *rad51*Δ double mutant and suppress the genomic instability phenotype of *tsa1*Δ mutants. Our results support a model in which the majority of spontaneous mutations that occur in wild-type strains are formed from lesions generated by ROS.

## Results

### ROS are the primary cause of genome instability in *tsa1*Δ cells

Tsa1 plays a key role in preventing mutations and genome rearrangements [13]. The mutator phenotype of *tsa1*Δ cells might be primarily due to their inability to reduce H_2_O_2_. The endogenous concentrations of H_2_O_2_ and alkyl hydroperoxides in wild-type and *tsa1*Δ strains have so far not been precisely determined, because available ROS sensors were not selective or sensitive enough [19]. However, several laboratories have detected a significant increased intracellular ROS levels in *tsa1*Δ strains [16], [20]–[23]. The conversion of this excess of endogenous H_2_O_2_ to hydroxyl radical through the Fenton reaction, believed to occur at or near DNA, could induce a variety of types of DNA damage [24]. Yet the increase of intracellular H_2_O_2_ in *tsa1*Δ cells versus wild-type cells was not high enough to change the nuclear and cytoplasmic GSH/GSSG redox state detectable by the genetically encoded redox sensor rxYFP [25] (Figure S1). It is generally accepted that the Can^R^ mutation rate in cells is indicative of the level of DNA damage, which itself follows the variations of ROS concentration. One way to decrease endogenous ROS concentrations is to grow yeast cells under anaerobiosis. The Can^R^ mutation rates of wild-type and *tsa1*Δ strains, grown in aerobic or anaerobic conditions, were measured by fluctuation analysis [26]. As shown in Table 1, the Can^R^ mutation rates were similar for wild-type cells grown under both conditions. An identical observation was published by Northam *et al*. [27]. In contrast, the Can^R^ mutation rate of the *tsa1*Δ strain decreased substantially under anaerobiosis and was the same as the Can^R^ mutation rate of the wild-type strain. This observation supports the view that scavenging intracellular H_2_O_2_ is one of the major cellular functions of Tsa1 that protects the nuclear genome from damage by ROS. These results suggest that ROS in *tsa1*Δ mutant grown under aerobic conditions is a major source of DNA damage underlying the formation of Can^R^ mutations. It may be not so surprising that the anaerobiosis does not reduce the basal level of Can^R^ mutation rate in the wild-type strain. In fact, anaerobiosis was shown to invoke a stress response in yeast cells [28], [29]. In addition, osmotic and DNA replication stress elicit mutagenesis [27], [30], [31]. Although the level of ROS is greatly reduced in anaerobic conditions and consequently the level of DNA damage generated, wild-type cells (as well as *tsa1*Δ cells) could sense and respond to anoxia. Indeed, hypoxia constitutes a profound cellular stress for mammalian cells that can promote genetic instability [32].

**Table 1:** Can^R^ mutation rates of wild-type and *tsa1*Δ strains grown in aerobic and anaerobic conditions.

		Mutation rates (× 10^−7^)	
Strain	Genotype	Aerobic growth	Anaerobic growth
GF4729	*wild-type*	2.13 (1.63–3.17)	3.03 (2.67–4.27)
GF5270	*tsa1*Δ	33.07 (25.16–64.02)	2.72 (2.43–4.24)

Mutation rates were determined as described in Material and Methods and were calculated each from 19 parallel cultures, using fluctuation analysis. 95% confidence intervals are indicated.

### Yap1 is not activated in *tsa1*Δ mutant

Yap1 is a redox sensitive transcription factor, considered as a central node in the oxidative stress response network [33]. Yap1 regulates the expression of several hundreds of genes (http://www.yeastgenome.org) among which are genes coding for enzymes with antioxidant properties, numerous oxido-reductases and transcription factors. In non-stressed wild-type cells, Yap1 exists mainly in its reduced form and is distributed equally throughout the cytoplasm and nucleus [34]. In response to oxidative stress, Yap1 rapidly forms two disulfide bonds, resulting in the inhibition of the Yap1 interaction with the nuclear export protein Crm1, and consequently its accumulation within the nucleus [34]. Thus, an excess of H_2_O_2_ in *tsa1*Δ cells may increase endogenous oxidative stress, and Yap1 should be oxidized. Published results have proven the difficulties to distinguish by Western blotting oxidized and reduced forms of Yap1 in W303 derived strains [35], [36]. As an alternative approach, we followed by qRT-PCR analysis the expression levels of several Yap1-target genes [8]–[10] in wild-type cells and in cells harbouring deletions *tsa1*Δ, *yap1*Δ or both (Table 2). Yap1 was found not activated in the *tsa1*Δ strain, as only small variations were observed between wild-type and *tsa1*Δ strains. These variations are in no way comparable to those produced by exogenous oxidative stress [37].

**Table 2:** Expression of Yap1-regulated oxido-reductase genes in *tsa1*Δ cells.

Strains	Genotype	*TSA2*	*TRX2*	*GRX2*	*GLR1*	*GSH1*	*CCP1*
GF4729	*wild-type*	1.0	1.0	1.0	1.0	1.0	1.0
GF5265	*tsa1*Δ	1.7	1.2	0.7	0.6	0.9	1.0
GF5384	*yap1*Δ	0.2	0.5	0.3	0.4	0.5	0.4
GF5381	*tsa1*Δ *yap1*Δ	1.2	0.5	0.4	0.4	0.25	0.9

The expression of six Yap1-regulated oxido-reductase genes was followed by quantitative RT-PCR. mRNA levels were calculated as ratios relative to that of the wild-type strain GF4729.

**Figure 1:**
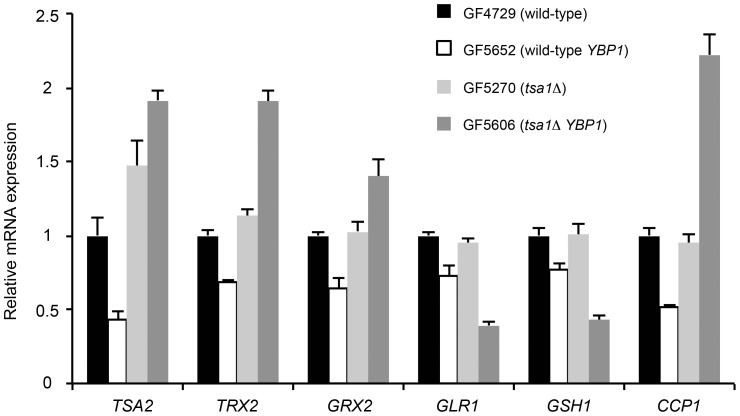
Quantitative RT-PCR analysis for indicated genes in GF4729 (wild-type *ybp1–2*), GF5652 (wild-type *YBP1*), GF5270 (*tsa1* Δ ***ybp1–2*** **) and GF5606 (** ***tsa1 YBP1*** **).** Quantitative RT-PCR analysis was performed as described in Material and Methods. mRNA levels of each gene were calculated as ratios relative to that of the wild-type *ybp1–2* strain (set as 1). The reported values are the mean of three independent experiments and the error bars represent the standard error of the mean.

What could be the cause of the non- or weak activation of Yap1 in *tsa1*Δ cells? It has been proposed that the Gpx3 sensor regulates the cellular H_2_O_2_ homeostasis in *S. cerevisiae* and is necessary for Yap1 activation. Gpx3 senses H_2_O_2_ and converts this signal into a cysteine-based redox cascade that culminates in oxidation of Yap1 [38], [39]. A third component, Ybp1 seems to be crucial for H_2_O_2_-induced Yap1 activation. Ybp1 forms an H_2_O_2_-induced complex with Yap1 and the transient Yap1-Gpx3 intermediate cannot be formed in the absence of Ybp1 [40], [41], although this mechanism is not fully understood. Strains harboring the W303 genetic background contain a mutated *YBP1* gene. In this context, Yap1 activation appears to be Gpx3-independent but requires Tsa1 [35]. The yeast strains used in this study are derived in part from W303 strain. We sequenced *YBP1* gene of GF4729 strain and identified all the mutations previously described in allele *ybp1–2* [35] (data not shown). Thus the absence of a functional Ybp1 protein might be responsible for the absence of Yap1 activation in the *tsa1*Δ strain. To test this hypothesis, we replaced the *ybp1–2* allele by the *YBP1* gene isolated from an S288c strain that is free of any mutation. We monitored by qRT-PCR analysis the expression level of six Yap1-target genes in GF4729 (wild-type, *ybp1–2*), GF5652 (wild-type, *YBP1*), GF5270 (*tsa1*Δ *ybp1–2*) and GF5606 (*tsa1*Δ, *YBP1*) strains. As shown in Figure 1, the expressions of these target genes were very similar between GF4729 (wild-type, *ybp1–2*) and GF5270 (*tsa1*Δ *ybp1–2*) strains. The expression of target genes changed within 2-fold between GF4729 (wild-type, *ybp1–2*) and GF5652 (wild-type, *YBP1*) strains, and between GF5270 (*tsa1*Δ *ybp1–2*) and GF5606 (*tsa1*Δ, *YBP1*) strains. Yet, these small variations did not affect significantly the Can^R^ mutations rates (Table 3). However, as shown by Okazaki and coworkers [35], it remains possible that a strain like GF5606 (*tsa1*Δ *YBP1*) can use preferentially the Ybp1/Gpx3 pathway to activate Yap1 in response to a strong exogenous oxidative stress.

**Table 3:** Can^R^ mutation rates of different mutant strains.

Strain	Genotype	Mutation rate × 10^−7^	95% confidence interval
GF4729	*wild-type*	4.20	3.98–4.82
GF5652	*wild-type YBP1*	3.76	3.44–4.40
GF5270	*tsa1*Δ	70.7	61.60–101.50
GF5606	*tsa1*Δ*YBP1*	65.57	52.76–77.23
GF5968	*tsa2*Δ	3.60	3.03–4.41
GF5965	*tsa1*Δ *tsa2*Δ	70.61	57.13–96.93
GF5505	*trr1*Δ	0.84	0.55–1.05
GF5499	*trr1*Δ *tsa1*Δ	2.15	1.88–2.78
GF6054	*trr1*Δ *tsa2*Δ	1.05	0.62–1.45
GF5967	*trr1*Δ *tsa1*Δ *tsa2*Δ	3.28	2.71–3.58
GF5668	*trx1*Δ *trx2*Δ	2.11	1.74–2.42
GF5898	*trr1*Δ *trx1*Δ *trx2*Δ	2.71	2.26–2.98
GF5674	*trx1*Δ *trx2*Δ *tsa1*Δ	5.75	5.21–7.05
GF6067	*trx1*Δ *trx2*Δ *tsa1*Δ *tsa2*Δ	5.87	4.94–7.25
GF5675	*rad51*Δ	38.26	30.50–46.42
GF5506	*trr1*Δ *rad51*Δ	9.41	7.30–12.35
GF6020	*trx1*Δ *trx2*Δ *rad51*Δ	80.54	65.35–97.57
GF5494	*trr1*Δ *tsa1*Δ *rad51*Δ	25.53	20.89–35.09
GF5959	*trr1*Δ *tsa1*Δ *tsa2*Δ *rad51*Δ	73.43	67.41–77.85

Each mutation rate was calculated from 19 to 57 independent cultures.

**Figure 2:**
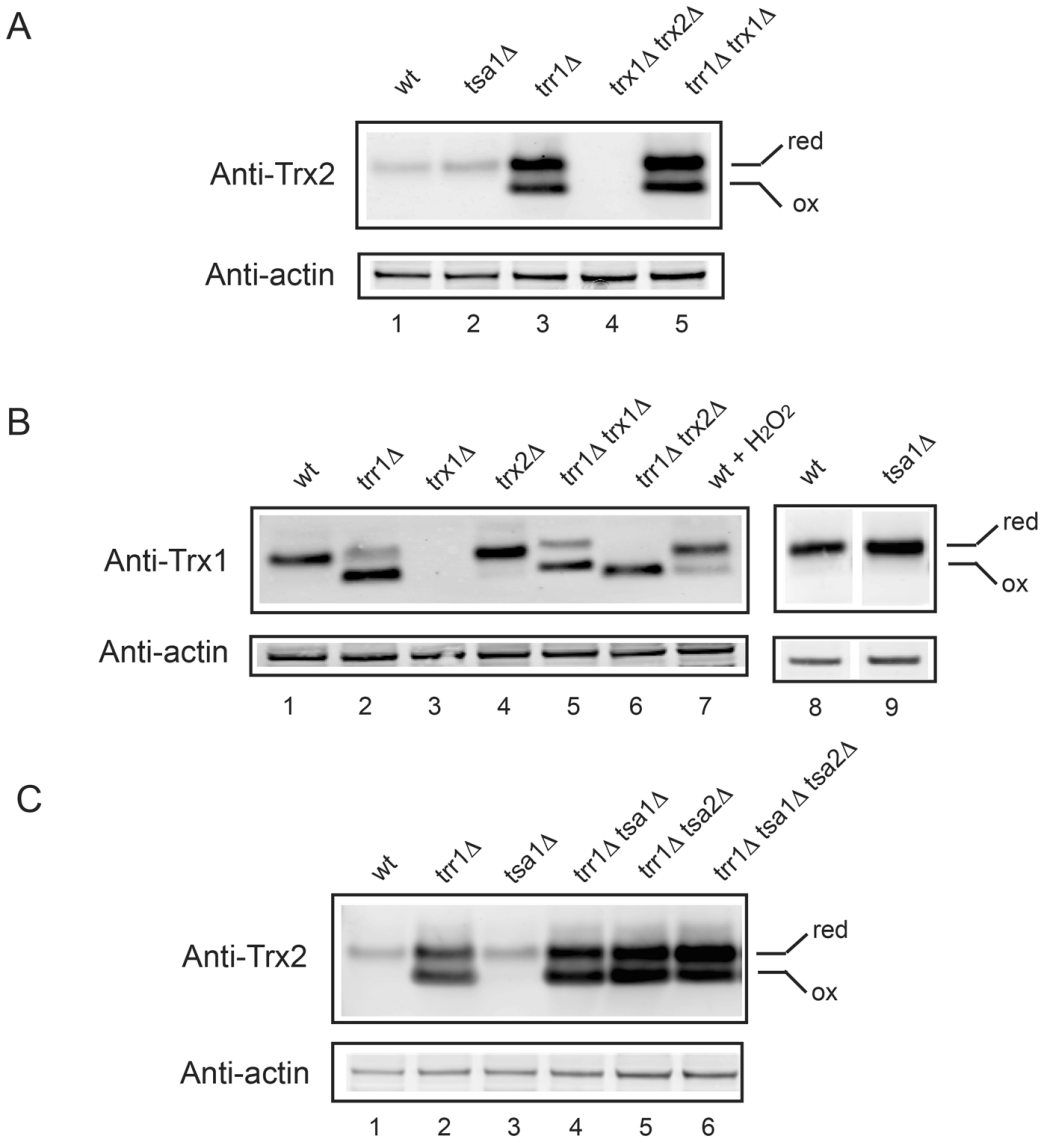
Oxidation state of Trx1 and Trx2 in different strains defective in maintaining redox homeostasis. Wild-type and mutated cells were processed for western blotting as described in Material and Methods. Proteins were detected with rabbit polyclonal anti-Trx2 (A, C) and anti-Trx1 (B) antibodies. Extracts from wild-type cells, treated with 0.5 mM H_2_O_2_ for 5 min in YPG, was loaded as a control (B, lane 7).

Thioredoxins are thought to be the physiological reducing agents and are responsible for the negative regulation of the Yap1 transcriptional activity [42]–[44]. In non-stressed wild-type cells, Trxs are kept almost exclusively in their reduced forms [42], [45], [46]. An increase of endogenous H_2_O_2_ concentration leads to the oxidation of Tsa1 that will be reduced in turn at the expense of reduced Trxs [4]. We estimated the redox state of Trxs in *tsa1*Δ cells using rabbit anti-Trx1 and Trx2 antibodies and found that both Trxs were in a reduced state in *tsa1*Δ cells as in wild type cells (Figure 2A, lanes 1, 2; Figure 2B, lanes 8, 9). Taken together, we propose that an increased endogenous concentration of H_2_O_2_ in *tsa1*Δ cells could result from the combined effect of the absence of Tsa1 itself and the inhibition of Yap1 activation by the reduced Trxs.

### Identification of a suppressor of the lethality of *tsa1*Δ *rad51*Δ double mutants

We previously observed that *tsa1*Δ *rad51*Δ double mutants were inviable under aerobic conditions but viable under anaerobic conditions [17], [18]. To screen for spontaneous mutations that rescue the viability of *tsa1*Δ *rad51*Δ double mutants under aerobic conditions, *tsa1*Δ *rad51*Δ cells were grown under anaerobic conditions, harvested and about 4×10^6^ cells were plated under aerobic conditions. Incubation under aerobic conditions resulted in massive cell death and the appearance of a small number of viable colonies. One viable colony from each of 20 independent experiments was selected and further characterized. These 20 strains each containing a potential suppressor mutation were then crossed with a *tsa1*Δ strain. Analysis of the meiotic segregants showed that the putative suppressor mutations (named *sup1* to *sup20*) were all single mutations, recessive and mapped at loci that were not linked to the starting *rad51*Δ mutation.

We used a cloning scheme employing the red/white sectored colony method described by Zhao and co-workers [47] to identify the suppressor genes. Briefly, *ADE2* and *ADE3* were disrupted in the *tsa1*Δ *rad51*Δ *sup1* strain and the plasmid p1591 (*CEN*, *URA3*, *TSA1*, *ADE3*) was introduced, yielding the strain GF5377. The presence of this plasmid, which is not needed for cell viability, confers a red color to the colonies on selective medium (SC-uracil). Cells growing in the absence of selection for this plasmid can spontaneously lose it and form red/white sectored or white colonies. As the *sup1* mutation was recessive, we expected that the introduction of a plasmid harboring the *SUP1* wild-type gene into strain GF5377 would result in red colonies since the resident plasmid p1591 (*CEN*, *URA3*, *TSA1*, *ADE3*) would now be required for growth. After transformation of strain GF5377 with a *CEN-LEU2* plasmid-based genomic library, candidate *SUP1* transformants were identified as solid red colonies on plates lacking leucine. After retesting 137 red colonies and then screening the resulting 21 strains for lack of growth on media containing 5-FOA, 7 plasmid-containing strains were obtained. Plasmids were rescued from these strains and sequenced which revealed that they each contained an ∼10 kb-long insert with an overlapping region of chromosome IV containing two full-length ORFs, *YDR352w* and *TRR1*. Subcloning of these two ORFs and further analysis showed that only *TRR1* could complement the *sup1* mutation in the GF5377 strain. We then determined the DNA sequence of the *TRR1* gene from each of the 20 independently isolated suppressor strains as well as that of the wild-type strain GF4729 revealing that each of the suppressor strains contained a mutation in *TRR1* (Table S4). To further confirm that inactivation of *TRR1* suppresses the inviability of the *tsa1*Δ *rad51*Δ double mutant, we deleted *TRR1* in a *tsa1*Δ/*TSA1 rad51*Δ/*RAD51* diploid strain and showed by tetrad analysis that the *TRR1* deletion restored the viability of double-mutant *tsa1*Δ *rad51*Δ in aerobic conditions (Table 4).

**Table 4:** *trr1*Δ deletion suppresses the lethality of *tsa1*Δ *rad51*Δ.

Segregant genotype	Number observed	Colony size
*tsa1*Δ *rad51*Δ *trr1*Δ	17	m
*tsa1*Δ *rad51*Δ*TRR1*	0	
*tsa1*Δ *RAD51 trr1*Δ	22	m
*tsa1*Δ*RAD51 TRR1*	18	G
*TSA1 rad51*Δ *trr1*Δ	11	p
*TSA1* *rad51*Δ *TRR1*	23	G
*TSA1* *RAD51* *trr1*Δ	15	p
*TSA1 RAD51* *TRR1*	17	G

Tetrad analysis of meiotic products of diploid strain *tsa1*Δ/*TSA1 rad51*Δ/*RAD51 trr1*Δ/*TRR1*. These three loci are unlinked. 40 tetrads were dissected. 20 segregants of each type were expected. Colony size: p <m <G.

*TRR1* encodes the cytosolic thioredoxin reductase, whose known major function is to reduce the oxidized forms of Trx1 and Trx2. Thioredoxin reductase and thioredoxins act as a disulfide reductase system and protect cells against both oxidative and reductive stress [4], [48]. Our results therefore suggest that deregulation of the thioredoxin redox system resulting from the *trr1* mutation may promote the mechanism suppressing lethality of *tsa1*Δ *rad51*Δ double mutants.

### The oxidized thioredoxins in *trr1*Δ mutants contribute to Yap1 activation

**Figure 3:**
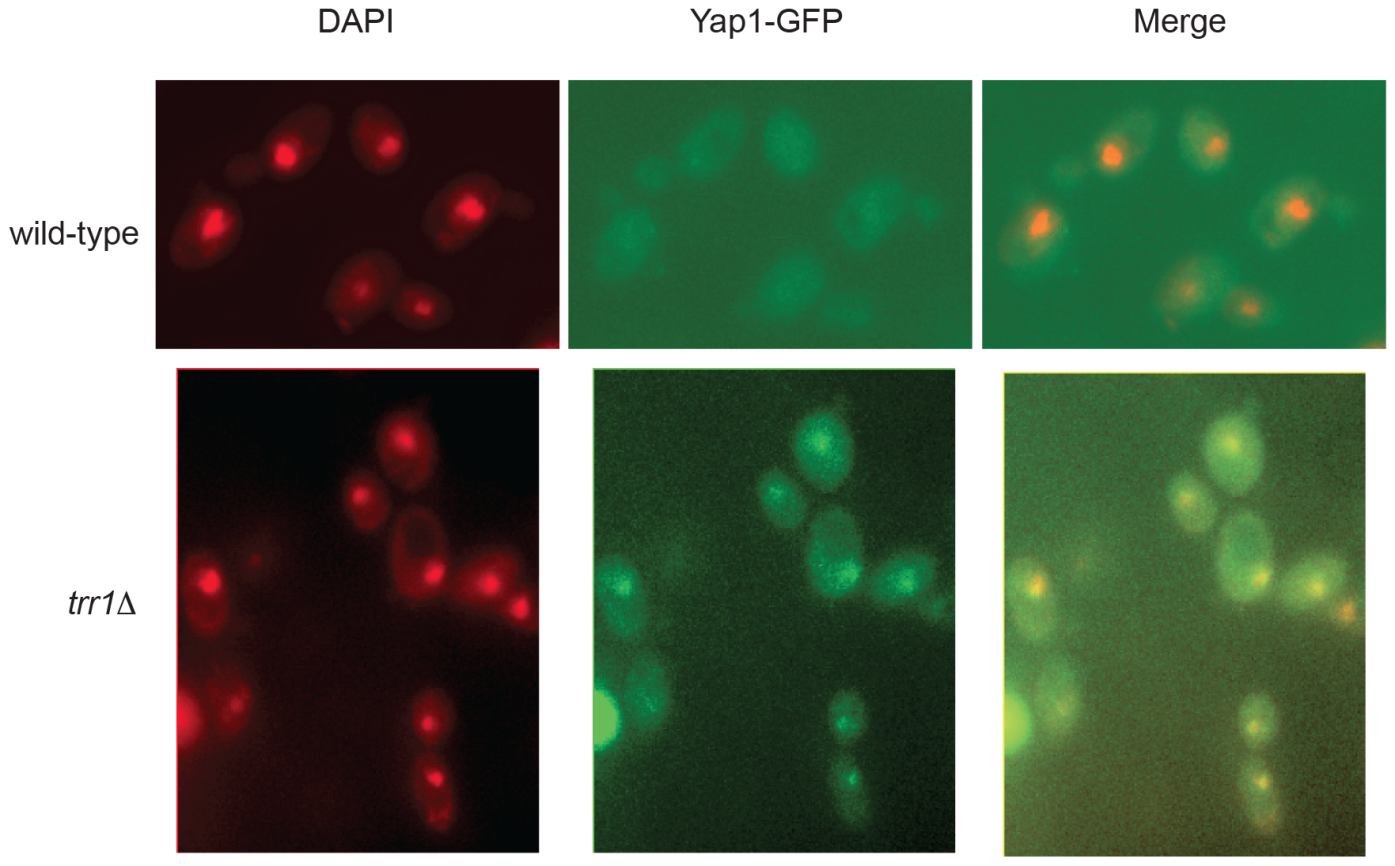
Localization of Yap1-GFP in *TRR1* and *trr1*Δ cells. Living cells in exponential phase expressing the Yap1-GFP fusion protein under the control of native Yap1 promoter and stained by DAPI were analyzed by fluorescence microscopy. Overlay image of DAPI and GFP signal (merge) are also shown.

It was previously reported that Yap1 is constitutively and partially oxidized and that the basal expression of Yap1 targets is elevated in *trr1*Δ mutants in absence of external stress [42], [49]. Besides, Trr1 inactivation enhances Msn2 response to H_2_O_2_ [50]. In fact, the transcriptional response to oxidative stress is known to depend on several transcription factors, including Yap1, Skn7, Msn2 and Msn4. Yap1 and Skn7 control independent but overlapping responses [8]; Msn2 and Msn4 mediate a transcriptional response which is common to many stress including oxidative stress [51]. We investigated whether a specific set of genes is transcriptionally regulated by Yap1/Skn7 and/or Msn2/Msn4 in *trr1*Δ mutants. The expressions of 13 genes encoding oxido-reductases were monitored by quantitative RT-PCR (Table 5). These genes include *TSA2* and *AHP1* whose expression is regulated by Yap1/Skn7 and Msn2/Msn4; *GLR1*, *GSH1*, *CCP1*, *YKL071w*, *TSA1*, *TRR1* and *TRX2* regulated by Yap1 or Yap1/Skn7; *CTT1*, *GRX1* and *GRX2* regulated by Msn2/Msn4, and finally *TRX1* which is not regulated by any of these transcription factors (http://www.yeastgenome.org). *YKL071w* is a gene of unknown function encoding a member of the dehydrogenase/reductase family [52]. Remarkably, the genes containing one or more Yap1 response elements (YRE) in their promoters were highly induced in the *trr1*Δ strain compared to the wild-type strain, especially *TSA2* (>600-fold), *TRX2* (30-fold), *CCP1* (30-fold) and *YKL071w* (>70-fold). As expected, *TRX1* was not up-regulated in *trr1*Δ strains. Interestingly, *CTT1* which is a typical target of Msn2/Msn4 transcription factor [50] was not induced in *trr1*Δ strains. Taken together, these results suggest that the over-expression of the oxido-reductases tested is mainly due to the activation of Yap1. Consistently, we observed that Yap1 accumulated in the nucleus of *trr1*Δ cells (Figure 3).

**Table 5:** Expression of Yap1-regulated or -independent oxido-reductases in different strains.

Strain	Genotype	Set	*TSA2*	*AHP1*	*CTT1*	*GRX1*	*GRX2*	*GLR1*	*GSH1*	*CCP1*	*YKL071w*	*TSA1*	*TRR1*	*TRX1*	*TRX2*
GF4729	*wild-type*	A	1.0	1.0	1.0	1.0	1.0	1.0	1.0	1.0	1.0	1.0	1.0	1.0	1.0
GF5630	*trx1*	A	1.0	1.7	1.4	0.9	1.0	1.1	1.3	1.2	0.7	1.5	1.3	NA	1.3
GF5643	*trx2*	A	1.2	1.6	1.4	1.1	0.9	1.1	1.2	1.2	1.0	1.3	1.3	1.2	NA
GF5668	*trx1 trx2*	B	10.4	3.9	1.2	3.9	4.2	3.3	3.9	8.0	8.8	3.0	4.5	NA	NA
GF5719	*trx1 trx2 tsa1*	B	10.3	3.6	1.5	3.2	2.5	1.5	2.1	4.2	7.2	NA	3.2	NA	NA
GF5898	*trr1 trx1 trx2*	B	15.8	5.6	0.6	3.3	2.6	2.2	3.4	11.7	11.2	4.2	NA	NA	NA
GF5911	*trr1 trx1 trx2 tsa1*	B	10.9	4.7	0.5	3.3	2.5	1.7	2.8	5.8	10.9	NA	NA	NA	NA
GF5505	*trr1*	C	657.4	6.1	1.4	2.5	7.3	2.5	4.2	29.1	71.7	7.0	NA	1.0	30.8
GF5888	*trr1 trx1*	C	1017.0	11.0	0.9	3.3	8.7	2.9	5.1	45.3	113.1	12.6	NA	NA	105.9
GF5899	*trr1 trx2*	C	1916.8	21.1	1.9	3.1	18.0	10.5	10.0	130.2	406.3	23.8	NA	3.2	NA
GF5909	*trr1 trx1 tsa1*	C	886.0	8.0	0.6	2.5	6.5	2.2	3.2	31.0	124.2	NA	NA	NA	39.0
GF5501	*trr1 tsa1*	C	370.0	4.2	1.3	1.9	4.4	1.7	2.4	12.2	38.0	NA	NA	0.9	20.1

**Figure 4:**
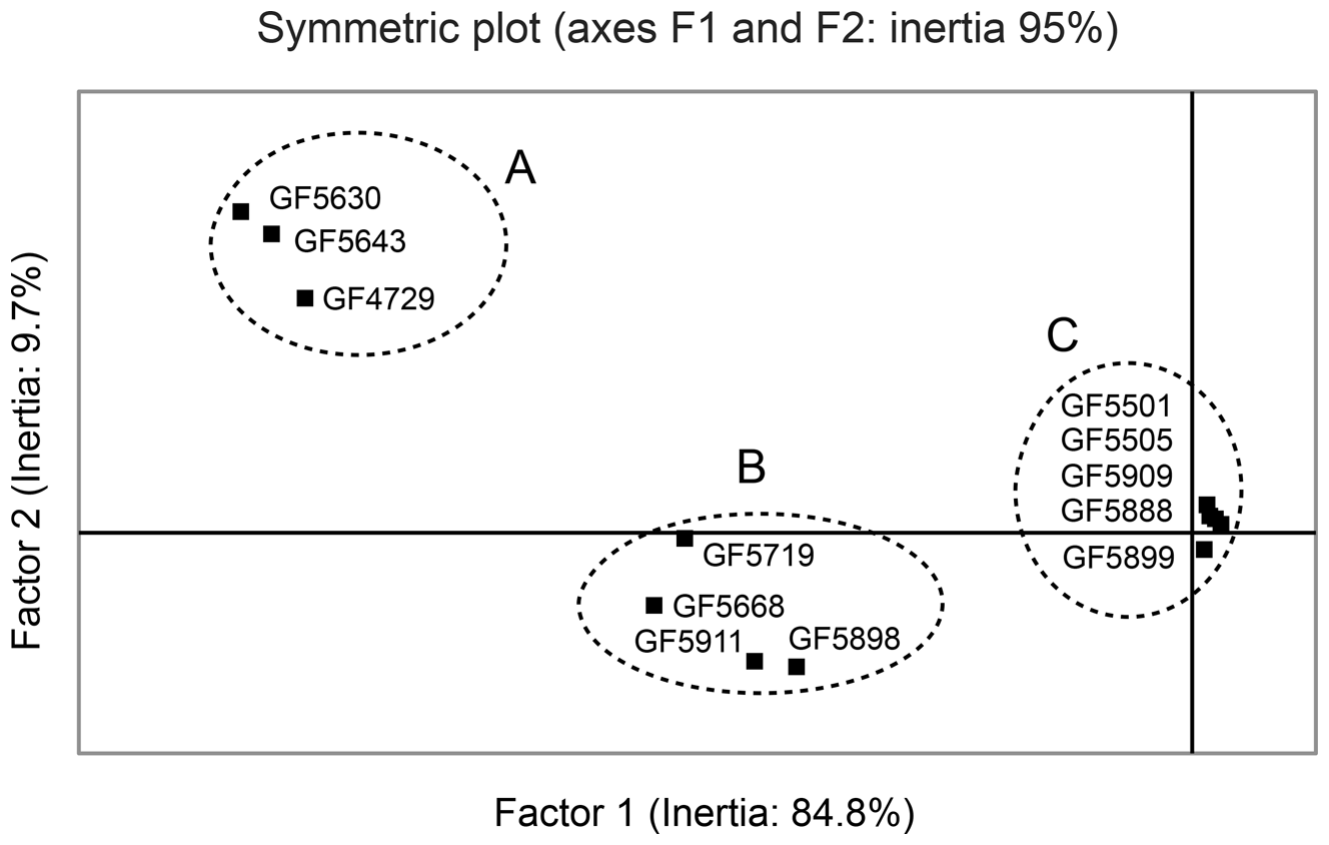
Correspondence analysis of qRT-PCR data. The correspondence analysis was performed using the program StatEL (Ad Science) and the quantitative RT-PCR data for genes *TSA2*, *AHP1*, *CTT1*, *GRX1*, *GRX2*, *GLR1*, *GSH1*, *CCP1* and *YKL071w* in 12 strains (Table 5). The percentage of inertia on the different axes is as follows: axis 1: 84.8%, axis 2: 9.7%, axis 3: 3.6%, axis 4: 0.8%, axis 5: 0.8%. Only the positions of the 12 yeast strains analysed are presented. They can be grouped in three distinct sets named A, B and C.

The expression of 13 oxido-reductase genes in 12 strains was measured by quantitative RT-PCR. mRNA levels were calculated as ratios relative to that of the wild-type strain GF4729. Yeast strains were ordered according to their membership to the three sets (A, B and C) highlighted by correspondence analysis (Figure 4). Genes *CTT1* and *TRX1* did not appear to be regulated by Yap1. NA, not applicable.

To address the question of how *trr1* mutations trigger the activation of Yap1, we investigated the involvement of the thioredoxins Trx1 and Trx2 in this process. Thioredoxins are the primary substrates of Trr1 and are thought to be the physiological reducing agents responsible for negatively regulating Yap1 activity [42]–[44]. In unstressed wild-type cells, Trxs are found almost exclusively in their reduced form [42], [45], [46]. We therefore used quantitative RT-PCR to monitor the expression level of the above defined 13 genes coding for oxido-reductases in a set of strains containing different combinations of *trr1*Δ, *trx1*Δ and *trx2*Δ deletion mutations (Table 5, Figure 4). Globally, the tested genes were not over-expressed in strains harboring either *trx1*Δ or *trx2*Δ individual deletion, they were moderately over-expressed in strains harboring the *trx1*Δ *trx2*Δ double deletion with or without *trr1*Δ, and they were highly over-expressed in strains harboring the *trr1*Δ deletion with either *trx1*Δ or *trx2*Δ. The double deletion *trr1*Δ *trx1*Δ or *trr1*Δ *trx2*Δ induced higher expression than *trr1*Δ with the highest effect in *trr1*Δ *trx2*Δ (Table 5, Figure 4). Different extents of Yap1 activation were most visible when comparing expression levels of genes *TSA2*, *AHP1*, *CCP1* and *YKL071w* among these mutants. The fact that the tested genes were significantly more activated in a *trr1*Δ, *trr1*Δ *trx1*Δ, or *trr1*Δ *trx2*Δ strain than in *trr1*Δ *trx1*Δ *trx2*Δ strain suggest that the presence of Trx1 or Trx2 largely contributes to the strong over-expression of the tested genes in *trr1*Δ cells. On the other hand, the moderate Yap1 activation in *trx1*Δ *trx2*Δ suggests the existence of some Trx1/Trx2-independent mechanisms.

We then analyzed the oxidation state of Trx1 and Trx2 in the wild-type, *trx1*Δ, *trx2*Δ, *trr1*Δ, *trr1*Δ *trx1*Δ and *trr1*Δ *trx2*Δ contexts using anti-Trx1 and anti-Trx2 antibodies. The anti-Trx1 antibody (kind gift from Dr. Grant) was prepared against Trx1 while slightly cross-reacting with Trx2 [45], [53]. The anti-Trx2 antibody was prepared against Trx2 (see Material and Methods). Western blots using anti-Trx1 antibody revealed that Trx1 was>90% reduced in wild-type and *trx2*Δ strains whereas no visible signal was observed with cell extracts from *trx1*Δ strains (Figure 2B, lanes 1, 4 and 3) (NB: The expression levels of *TRX2* and *TRX1* in *trx1*Δ and *trx2*Δ strains respectively, were similar to that in wild-type (Table 5)). The same anti-Trx1 antibody revealed that Trx1 is>90% oxidized in *trr1*Δ *trx2*Δ cell extracts (Figure 2B, lane 6) whereas oxidized (70–75%) and reduced (25–30%) forms were detected in *trr1*Δ and *trr1*Δ *trx1*Δ extracts (Figure 2B, lanes 2 and 5), which indicates that anti-Trx1 antibody cross-reacts with Trx2 when Trx2 is over-expressed (Table 5). Using anti-Trx2 antibody, western blots of *trr1*Δ *trx2*Δ cell extracts yielded no signal, (Figure 2A, lane 4) whereas in *trr1*Δ and *trr1*Δ *trx1*Δ cell extracts, Trx2 was at least 10-times more expressed than in the wild type strain and 40–50% Trx2 was in its oxidized form (Figure 2A, lanes 3 and 5). The fact that Trx2 is not completely oxidized in *trr1*Δ cells implies existence of an alternative electron donor. Indeed, recent data suggest that GSH, directly or indirectly, is able to reduce oxidized Trxs [54], [55].

These results raise the question of whether the over-expression of the Yap1-targeted genes in *trr1*Δ cells is due to a high concentration of oxidized Trxs or conversely the decreased level of reduced Trxs, since the latter form may impede Yap1 activation. If thioredoxins regulate Yap1 activity by the inhibitory effect of their reduced forms, then Yap1 activity in *trx1*Δ *trx2*Δ mutant should be at least similar, if not higher, than in *trr1*Δ mutants, which was not the case (Table 5). Hence we conclude that, very probably, the oxidized thioredoxins intervene in Yap1 activation in *trr1*Δ *trr1*Δ *trx1*Δ or *trr1*Δ *trx2*Δ cells, directly or indirectly. Consistently, Yap1 activation is the strongest in GF5899 strain (*trr1*Δ *trx2*Δ) (Table 5) in which Trx1 is more than 90% oxidized (Figure 2). In *trr1*Δ *tsa1*Δ cells, Yap1 is about 40% less activated than in *trr1*Δ cells (Table 5) suggesting that Tsa1 might play a role in Yap1 oxidation [36].

### The absence of Trr1 suppresses mutagenesis in *tsa1*Δ and *rad51*Δ strains

Loss of Trr1 can fully rescue the viability of *tsa1*Δ *rad51*Δ double mutants and is associated with over-expression of Yap1-controlled oxido-reductases. We reasoned that over-expression of oxido-reductases may reduce significantly intracellular ROS level and therefore suppress genomic instability of *tsa1*Δ and/or *rad51*Δ strains. As previously reported, *TSA1* deletion resulted in significant increase in Can^R^ mutation rate, 70.70×10^−7^, compared to the wild-type strain that was 4.20×10^−7^ (Table 3). Can^R^ mutation rate for a *rad51*Δ was 38.26×10^−7^. The synthetic lethality between *tsa1*Δ and *rad51*Δ may mean that cells cannot cope with toxic levels of DNA damage in a *tsa1*Δ *rad51*Δ haploid strain grown in aerobic conditions. Furthermore, the Can^R^ rate in a *trr1*Δ *tsa1*Δ strain was 2.15×10^−7^ that is a 33-fold reduction compared to a *tsa1*Δ single mutant and is lower than the Can^R^ mutation rate of the wild-type strain. Similarly but to a lesser extent, Can^R^ mutation rate for a *rad51*Δ *trr1*Δ mutant was 9.41×10^−7^ that is a 4-fold reduction compared to *rad51*Δ single mutant. These different effects of *trr1*Δ on *tsa1*Δ and *rad51*Δ may suggest that *tsa1*Δ -induced increased mutagenesis results from increased ROS accumulation and can be efficiently attenuated by over-expression of oxido-reductases while *rad51*Δ -induced mutagenesis may be only partially linked to ROS accumulation. The Can^R^ mutation rate in *tsa1*Δ *rad51*Δ *trr1*Δ strain was 25.53×10^−7^, corresponding to a multiplicative effect of mutations of *tsa1*Δ *trr1*Δ and *rad51*Δ *trr1*Δ (2.15×9.41). Taken together, our results suggest that *trr1*Δ rescues the viability of *tsa1*Δ *rad51*Δ double mutants through suppression of *tsa1*Δ and *rad51*Δ -induced mutagenesis that is achieved by redox regulated over-expression of oxido-reductases.

Interestingly, the Can^R^ mutation rate in a *trr1*Δ strain was 0.84×10^−7^, 5-fold lower than in a wild-type strain. Due to the over-expression of oxido-reductases, the ROS level in *trr1*Δ cells should be very low. We can estimate the respective contribution of Tsa1 and Tsa2 in ROS reduction in the *trr1*Δ context by comparing the Can^R^ mutations rates. The mutation rates of *trr1*Δ *tsa1*Δ, *trr1*Δ *tsa2*Δ and *trr1*Δ *tsa1*Δ *tsa2*Δ strains were 2.15×10^−7^, 1.05×10^−7^ and 3.28×10^−7^, respectively. These data suggest that the over-expression of Tsa1 and Tsa2 in *trr1*Δ cells plays a role in reducing ROS level, but others oxido-reductases are also involved.

We analyzed the meiotic products of diploid strain *trr1*Δ/*TRR1 tsa1*Δ/*TSA1 rad51*Δ/*RAD51 tsa2*Δ/*TSA2*. Tsa2 was not essential for the viability of a mutant carrying the triple deletion *trr1*Δ *tsa1*Δ *rad51*Δ Table 6. Nevertheless, Tsa1 and Tsa2 contributed to suppress the Can^R^ mutation formation of *trr1*Δ *rad51*Δ (9.41×10^−7^) since Can^R^ mutation rate was 25.53×10^−7^ and 74.45×10^−7^ in *trr1*Δ *tsa1*Δ *rad51*Δ and *trr1*Δ *tsa1*Δ *tsa2*Δ *rad51*Δ strains respectively. Viability of *trr1*Δ *tsa1*Δ *tsa2*Δ *rad51*Δ could be attributed to the over-expression of other oxido-reductases. To further clarify the relationship between Tsa1/Tsa2 and thioredoxins, we analyzed the oxidation state of Trx2 in *trr1*Δ, *trr1*Δ *tsa1*Δ, *trr1*Δ *tsa2*Δ and *trr1*Δ *tsa1*Δ *tsa2*Δ strains using anti-Trx2 antibody. Constant ratios of the reduced and oxidized forms of Trx2 were found in *trr1*Δ, *trr1*Δ *tsa1*Δ and *trr1*Δ *tsa2*Δ cells (Figure 2C, lanes 2, 4, 5), whereas the ratio was slightly increased in the *trr1*Δ *tsa1*Δ *tsa2*Δ strain (Figure 2C, lane 6), suggesting that the reduced form of Trx2 is not consumed more by Tsa1 or Tsa2 in *trr1*Δ strains than in wild-type strains.

**Table 6:** Effect of *tsa2*Δ deletion on the lethality of *tsa1*Δ *rad51*Δ *trr1*Δ.

Segregant genotype	Number observed
*tsa1*Δ *tsa2*Δ *rad51*Δ *trr1*Δ	47
*tsa1*Δ *tsa2*Δ *rad51*Δ *TRR1*	1
*tsa1*Δ *tsa2*Δ *RAD51 trr1*Δ	54
*tsa1*Δ *tsa2*Δ *RAD51 TRR1*	53
*tsa1*Δ *TSA2 rad51*Δ *trr1*Δ	42
*tsa1*Δ *TSA2 rad51*Δ *TRR1*	0
*tsa1*Δ *TSA2 RAD51 trr1*Δ	44
*tsa1*Δ *TSA2 RAD51 TRR1*	48
*TSA1 tsa2*Δ *rad51*Δ *trr1*Δ	37
*TSA1 tsa2*Δ *rad51*Δ *TRR1*	48
*TSA1 tsa2*Δ *RAD51 trr1*Δ	34
*TSA1 tsa2*Δ *RAD51 TRR1*	46
*TSA1 RAD51 rad51*Δ *trr1*Δ	34
*TSA1 RAD51 rad51*Δ *TRR1*	57
*TSA1 RAD51 RAD51 trr1*Δ	43
*TSA1 RAD51 RAD51 TRR1*	55
expected	55

Tetrad analysis of meiotic products of diploid strain *tsa1*Δ/*TSA1 tsa2*Δ/*TSA2 rad51*Δ/*RAD51 trr1*Δ/*TRR1*. These four loci are unlinked. 220 tetrads were dissected. 55 segregants of each genotype were expected.

As *trx1*Δ *trx2*Δ double deletion exhibits only a moderate effect in inducing overexpression of oxido-reductases, we determined whether this double deletion has a significant effect on the mutation formation in *tsa1*Δ and *rad51*Δ strains and is capable of rescuing viability of *tsa1*Δ *rad51*Δ mutants. The Can^R^ mutation rate of a *trx1*Δ *trx2*Δ strain was 2.11×10^−7^ (Table 3), that is 2-fold less than in a wild-type strain. The Can^R^ rate in a *trx1*Δ *trx2*Δ *tsa1*Δ strain was 5.75×10^−7^ that is a 12-fold reduction compared to a *tsa1*Δ strain but is higher than that in *trr1*Δ *tsa1*Δ strain (2.15×10^−7^). Surprisingly, the Can^R^ mutation rate of the *rad51*Δ *trx1*Δ *trx2*Δ strain, 80.54×10^−7^, is 2-fold higher compared to *rad51*Δ strain. Therefore, the inactivation of *TRR1* attenuates deleterious mutagenic consequences of both *tsa1*Δ and *rad51*Δ deletions, while the *trx1*Δ *trx2*Δ double deletion only reduces the mutagenic effect linked to *tsa1*Δ but increases DNA damage in *rad51*Δ. Consequently, the *tsa1*Δ *rad51*Δ *trx1*Δ *trx2*Δ cells may not be able to cope with the high level of DNA damage still formed. Indeed, we determined whether *trx1*Δ *trx2*Δ might rescue the synthetic lethality of a strain bearing *tsa1*Δ *rad51*Δ We analyzed the meiotic products of diploid strain *tsa1*Δ/*TSA1 rad51*Δ/*RAD51 trx1*Δ*/TRX1 trx2*Δ*/TRX2* and obtained only 10 *tsa1*Δ *rad51*Δ *trx1*Δ *trx2*Δ segregants instead of the 35 expected (Table 7). The severe slow growth of these segregants prevented us to assess their Can^R^ mutation rates. Therefore, the different effects resulting from *TRR1* deletion or *TRX1* and *TRX2* double deletion seem related to the expression levels of oxido-reductases in these different strains.

**Table 7:** Effect of *trx1*Δ *trx2*Δ double deletion on the lethality of *tsa1*Δ *rad51*Δ.

Segregant genotype	Number observed	Colony size
*tsa1*Δ *rad51*Δ *trx1*Δ *trx2*Δ	10	pp
*tsa1*Δ *rad51*Δ *trx1*Δ *TRX2*	1	
*tsa1*Δ *rad51*Δ *TRX1 trx2*Δ	0	
*tsa1*Δ *rad51*Δ *TRX1 TRX2*	0	
*tsa1*Δ *RAD51 trx1*Δ *trx2*Δ	28	G
*tsa1*Δ *RAD51 trx1*Δ *TRX2*	29	G
*tsa1*Δ *RAD51 TRX1 trx2*Δ	39	G
*tsa1*Δ *RAD51 TRX1 TRX2*	38	G
*TSA1 rad51*Δ *trx1*Δ *trx2*Δ	28	p
*TSA1 rad51*Δ *trx1*Δ *TRX2*	32	m
*TSA1 rad51*Δ *TRX1 trx2*Δ	34	G^–^
*TSA1 rad51*Δ *TRX1 TRX2*	38	m
*TSA1 RAD51 trx1*Δ *trx2*Δ	45	m
*TSA1 RAD51 trx1*Δ *TRX2*	28	m^+^
*TSA1 RAD51 TRX1 trx2*Δ	31	G
*TSA1 RAD51 TRX1 TRX2*	31	G

Tetrad analysis of meiotic products of diploid strain *tsa1*Δ/*TSA1 rad51*Δ/*RAD51 trx1*Δ/*TRX1 trx2*Δ/*TRX2*. These four loci are unlinked. 140 tetrads were dissected. 35 segregants of each genotype were expected. Colony size: pp <p <m <m^+^ <G^–^ <G.

### Impact of dNTP levels on the Can^R^ mutation rates in *trr1*Δ and *trx1*Δ *trx2*Δ cells

**Figure 5:**
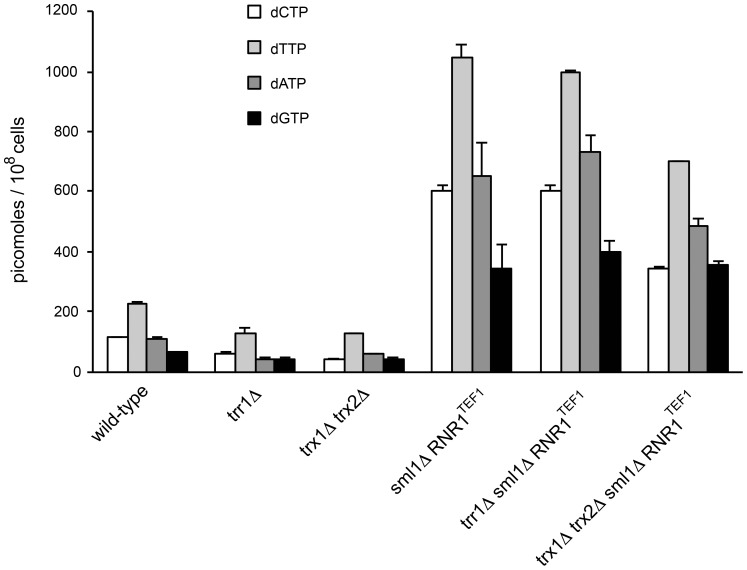
dNTP concentrations in yeast strains. dNTP levels were measured in wild-type, *trr1*Δ *trx1*Δ *trx2*Δ, *sml1*Δ *RNR1^TEF1^*, *trr1*Δ *sml1*Δ *RNR1^TEF1^* and *trx1*Δ *trx2*Δ *sml1*Δ *RNR1^TEF1^* strains. Values are the average of two experiments and error bars represent the standard error of the mean.

Thioredoxins are physiologically relevant electron donors for ribonucleotide reductase (Rnr) during DNA precursor synthesis. Yeast cells lacking both cytoplasmic thioredoxins (*trx1*Δ *trx2*Δ) accumulate oxidized Rnr1, have a low dNTPs pool and a 3-fold longer S phase than wild-type cells [5]. Trx1 and Trx2 was constitutively strongly oxidized in the absence of Trr1. Furthermore, Trx2 expression was at least 10-times higher in the *trr1*Δ mutants than in wild-type cells and about 40–50% of Trxs was in oxidized form (Figure 2). These changes may directly or indirectly interfere with the activity of ribonucleotide reductase and affect dNTP synthesis. To estimate the impact of dNTP concentration on the Can^R^ mutation rates in the wild-type, *trr1*Δ and *trx1*Δ *trx2*Δ contexts, we increased ribonucleotide reductase activity by deleting *SML1* gene whose product is an ribonucleotide reductase inhibitor [56], and by placing *RNR1* gene under the control of the strong *TEF1* promoter. As shown in Figure 5 and Table S5, the dNTP levels of *trr1*Δ and *trx1*Δ *trx2*Δ were approximately 2-fold lower than that of wild-type cells grown asynchronously in YPD medium. *SML1* deletion and *RNR1* overexpression increased dNTP level from 1 to 5.22 in the wild-type cells (basal dNTP level in the wild type was set as 1), from 0.50 to 5.52 in the *trr1*Δ and from 0.52 to 3.93 in the *trx1*Δ *trx2*Δ context. In the *trr1*Δ and *trx1*Δ *trx2*Δ contexts, large increases of dNTP concentration produced 2.8-fold and 5.5-fold increases in the Can^R^ mutation rate, respectively (Table 8). In contrast, the increase of dNTP concentration had almost no effect in wild-type cells. In *E. coli*, lower dNTP levels correlate with a decreased spontaneous point mutation rate while higher dNTP levels correlate with an increased rate [57], [58]. In *S. cerevisiae,* such correlations were also observed in mutants affecting DNA damage checkpoints, RNR, replication factors or DNA repair components [14], [59], [60]. Taken together, dNTP fluctuations have more pronounced impact on the mutation formation in mutant cells than in wild-type cells.

**Table 8:** Impact of dNTP levels on the Can^R^ mutation rates.

Strain	Genotype	Yap1 activation	dNTP concentration*	Can^R^ rate (× 10^−7^)
GF4729	*wild-type*	no	1	4.20 (3.98–4.82)
GF5270	*tsa1*Δ	no	2.5**	70.70 (61.60–101.50)
GF5505	*trr1*Δ	strong	0.50	0.84 (0.55–1.05)
GF5499	*trr1*Δ *tsa1*Δ	strong	ND	2.15 (1.88–2.78)
GF5668	*trx1*Δ *trx2*Δ	moderate	0.52	2.11 (1.74–2.42)
GF5674	*trx1*Δ *trx2*Δ *tsa1*Δ	moderate	ND	5.75 (5.21–7.05)
GF6080	*sml1*Δ *RNR1^TEF1^*	ND	5.22	5.04 (4.34–6.13)
GF6084	*trr1*Δ *sml1*Δ *RNR1^TEF1^*	ND	5.52	2.35 (2.08–2.54)
GF6129	*trx1*Δ *trx2*Δ *sml1*Δ *RNR1^TEF1^*	ND	3.93	11.53 (9.55–12.25)

*dNTP concentration in mutant strains relative to wild-type cells (set to be 1).

** Davidson et al. 2012; ND, not determined.

**Figure 6:**
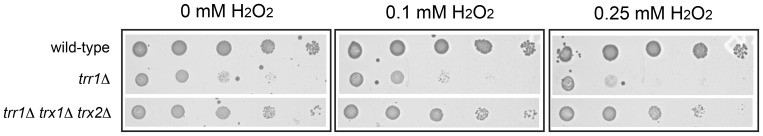
Sensitivity of wild-type, *trr1*Δ and *trr1*Δ *trx1*Δ *trx2*Δ strains to H_2_O_2_. Equal numbers of cells were serially diluted (10-fold dilution) and spotted onto SC medium containing indicated concentrations of H_2_O_2_. Yeasts growth was assessed after 4 days at 30°C.

Low levels of dNTP in *trr1*Δ and *trx1*Δ*trx2*Δ mutants compared to wild-type strains suggest that the decrease of Can^R^ mutation rate in these mutants may be due to the combined effects of Yap1 activation and dNTP pool reduction. To estimate their relative contributions, we propose a tentative model which links the quantity of endogenous DNA lesion and the cellular dNTP concentration to the production of Can^R^ mutations (Text S1). This model predicts that ROS level in *trr1*Δ is about 21 times lower than that in *trx1*Δ *trx2*Δ cells. These estimations are consistent with the observation above that the oxido-reductasesinvolved in the reduction of ROS are more actively synthesized in *trr1*Δ than in *trx1*Δ *trx2*Δ (Table 5, Table S6). On the other hand, the model predicts that the impact of dNTP concentration on the Can^R^ mutation rate is dependent on the level of lesions produced by ROS and processed by translesion polymerases. As more lesions are present in *trx1*Δ *trx2*Δ cells than in *trr1*Δ cells, changes in dNTP concentration will have more impact in the generation of Can^R^ mutations in *trx1*Δ *trx2*Δ than in *trr1*Δ cells. We note that these proposals seem to contradict the fact that *trr1*Δ cells are more sensitive to hydrogen peroxide than wild-type cells [45], [49]. We compared the growth of wild-type, *trr1*Δ and *trr1*Δ *trx1*Δ *trx2*Δ strains on SC plates containing 0.10 and 0.25 mM H_2_O_2_ (Figure 6). In these H_2_O_2_ concentration ranges, *trr1*Δ *trx1*Δ *trx2*Δ strain was almost as resistant as the wild-type strain, pointing to the possibility that the sensitivity of the *trr1*Δ cells is primarily due to the accumulation of toxic oxidized thioredoxins which disturb the cellular redox homeostasis, as previously suggested [4].

## Discussion

Among the five *S. cerevisiae* genes encoding peroxiredoxins, deletion of *TSA1* causes the accumulation of a broad spectrum of mutations. The mutator phenotype of *tsa1*Δ cells might be primarily due to their inability to reduce H_2_O_2_. The endogenous concentrations of H_2_O_2_ and alkyl hydroperoxides in wild-type and *tsa1*Δ strains have so far not been precisely determined because of the lack of the selective, quantitative and sensitive ROS probes [19]. Nevertheless, several laboratories report an increased intracellular ROS in *tsa1*Δ strains [16], [20]–[23]. Importantly, Can^R^ mutation rate of a *tsa1*Δ strain decreases substantially under anaerobiosis, further suggesting that the increase of intracellular H_2_O_2_ concentration in *tsa1*Δ cells is the primary cause of the genome instability of these cells under aerobiosis. In such conditions, mitochondria and other sources of ROS (peroxisome and endoplasmic reticulum) generate less ROS, due to the restricted availability of O_2_ which is a substrate for electrons [61], [62]. Consistently, loss of Trr1 function reduces substantially the Can^R^ mutation rate of *tsa1*Δ strain, most probably through over-expression of Yap1-regulated oxido-reductases that reduce substantially ROS-associated DNA damages. On the other hand, the associated expansion of the dNTP pool in *tsa1*Δ cells also contributes to its mutator phenotype [16], [60]. The excess of ROS in *tsa1*Δ cells increases the level of DNA lesions that in turn activates the DNA damage checkpoint pathway leading to the induction of RNR and the consequent overproduction of dNTPs. This double effect of increased ROS accumulation: elevation of DNA lesions level and stimulation of ribonucleotide reductase activity, may explain why the *tsa1*Δ mutant deviates from a correlation linking the increased dNTP level with increased spontaneous mutagenesis [60]. All these evidences support the view that the increase of intracellular H_2_O_2_ concentration in *tsa1*Δ cells is the primary cause of the genome instability of these cells under aerobiosis.

The transcription factor Yap1 is a central regulator of the response to oxidative stress in *S. cerevisiae*. In yeast cells with functional Gpx3 and Ybp1 proteins, Gpx3 senses exogenous added H_2_O_2_ and mediates H_2_O_2_ signal into a cysteine-based redox cascade that culminates in the oxidation of Yap1 [38], [39]. In strains harboring the W303 genetic background, the *YBP1* gene is mutated and nonfunctional, and Yap1 activation appears to be Gpx3-independent but requires Tsa1 [35]. Thioredoxins are physiological reducing agents and are responsible for the negative regulation of the Yap1 transcription factor activity [42]–[44]. Indeed, thioredoxins should be able to reduce the activated disulfide form of Yap1, preventing its translocation to nucleus or facilitating its exit to cytoplasm. In the present study, we have shown that the loss of Trr1 induces Yap1 accumulation in nucleus and over-expression of a set of Yap1-regulated oxido-reductases. Several evidence strongly suggest that oxidized thioredoxins are a key activator of Yap1 in *trr1*Δ mutants. First, as thioredoxins reduce the activated disulfide form of Yap1, highly oxidation of thioredoxins in *trr1*Δ cells should prevent their capacity to reduce oxidized Yap1 and facilitate its accumulation in nucleus. Secondly, we observed that a set of Yap1-regulated genes is significantly more over-expressed in a *trr1*Δ, *trr1*Δ *trx1*Δ, or *trr1*Δ *trx2*Δ strain than in *trr1*Δ *trx1*Δ *trx2*Δ strain, suggesting that the presence of Trx1 and/or Trx2 largely contributes to the strong activation of the tested genes in *trr1*Δ cells. Furthermore, as Yap1 activity in *trx1*Δ *trx2*Δ mutants is lower than in *trr1*Δ mutants, this suggests that Yap1 activation in *trr1*Δ cells is due to a high concentration of oxidized thioredoxins but not the decreased level of reduced thioredoxins. In addition to the thioredoxins, Tsa1 contributes also to the Yap1 activation, as expression levels of Yap1-regulated genes are significantly lower in *trr1*Δ *tsa1*Δ than in *trr1*Δ mutants. Finally, a moderate but significant Yap1 activation is noteworthy in *trx1*Δ *trx2*Δ cells suggesting the existence of some Trx1/Trx2-independent mechanisms. The precise mechanisms in which oxidized thioredoxins and Tsa1 or other factors are involved in Yap1 oxidation and activation remain to be determined in the contexts of present mutant strains.

The *tsa1*Δ *rad51*Δ double mutant is not viable under aerobic conditions but viable under anaerobic conditions [18]. This observation suggests that a large excess of DNA lesions produced by ROS are the cause of lethality in the absence of efficient recombination repair. In searching for spontaneous suppressors of synthetic lethality of the *tsa1*Δ *rad51*Δ double mutant, we identified that loss of thioredoxin reductase Trr1 rescues their viability. This unexpected effect seems to result from the strong impact of *TRR1* deletion on mutation formation. Additional deletion of *TRR1* in *tsa1*Δ mutants reduces substantially the Can^R^ mutation rate of *tsa1*Δ strain (33-fold), and to a less extend, of *rad51*Δ strain (4-fold). We reason that the synthetic lethality of *tsa1*Δ *rad51*Δ double mutant may result from excessive DNA damage. Substantial reduction of *tsa1*Δ and *rad51*Δ mutation rates in the *trr1*Δ context rescues the viability of the double mutant.

**Figure 7:**
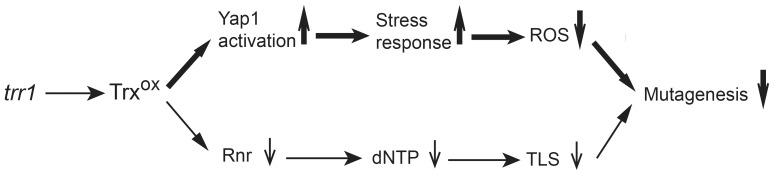
Schematics for *trr1*Δ-mediated decrease of Can^R^ mutation. Loss-of-function of thioredoxin reductase Trr1 results in constitutive oxidation of thioredoxins, which leads to suppression of mutation formation through two distinct mechanisms. Yap1 activation and consequent over-expression of Yap1-regulated oxido-reductases play a major role in reducing mutagenesis. TLS, translesion DNA synthesis.

In conclusion, we propose that the *trr1*Δ -induced mutation suppression effect results from at least two distinct mechanisms both mediated by oxidized thioredoxins (Figure 7). Constitutive oxidation of thioredoxins strongly activates Yap1, induces over-expression of a set of Yap1-regulated oxido-reductases with antioxidant properties that ultimately reduces substantially intracellular ROS concentration and consequently ROS associated DNA damages. Besides, the oxidized thioredoxins may directly interfere with the activity of ribonucleotide reductase, leading to a reduction in the dNTPs synthesis and the translesion DNA synthesis (TLS), further reducing the mutagenesis. Although Yap1 could regulate the expression of several chaperones and components involved in post-replication repair (http://www.yeastract.com), it remains to determine whether these elements could contribute significantly to suppress the mutator phenotype of *tsa1*Δ mutants. In combination with the model described in Text S1, we strengthen the view that in yeast cells, the large majority of spontaneous mutations originates from ROS-induced lesions.

## Materials and Methods

### Media

Complete medium (YPD) contained 1% (w/v) yeast extract, 1% (w/v) bactopeptone, 2% (w/v) glucose. Synthetic complete medium (SC) with 2% glucose was prepared as described by Sherman [63]. SPO2 sporulation medium contained 0.5% yeast extract, 0.5% bactopeptone, 2% potassium acetate. Canavanine-resistant mutants (Can^R^) caused by inactivation of the *CAN1* gene were selected on SC-arginine dropout plates containing 60 mg/liter canavanine. 5-fluoroorotic acid (5-FOA) was used at 1 g/liter in SC medium.

### Plasmid and strain constructions

The constructions of plasmids and strains used in this work are described in Text S2 and Text S3. All yeast strains used in this study are listed in Table S1. All oligonucleotides used for plasmid and strain constructions are listed in Table S2.

### Fluctuation analysis

The rate of accumulation of Can^R^ mutations in cell populations was determined by fluctuation analysis [26]. 2-ml cell cultures were incubated under aerobic conditions at 30°C for 3 days with agitation. The number of mutant cells per culture among 19–57 parallel cultures was calculated and the median of the distribution was used to determine the mutation rate of a given strain. The 95% confidence intervals for a median rate were calculated according to Huang and Kolodner [17].

### Anaerobic growth conditions

YPD medium was supplemented with Tween 80 and ergosterol to a final concentration of 1.32 g/liter and 6.75 mg/liter, respectively. For determining mutations rates, 2-ml cell cultures were placed in an 8-litre airtight jar containing 3 disposable hydrogen- and carbon dioxide-generating envelopes (BD BBL GasPak Plus, Becton Dickinson) and grown anaerobically at 30°C for 5 days without agitation. Anaerobic conditions were monitored with the redox indicators Anaerotest (Merck, Darmstadt) placed inside the airtight jar. As controls, cell cultures were grown under the same conditions except aerobically.

### Quantitative RT-PCR

Wild-type and mutated strains were grown to mid-exponential phase (OD 0.5, 600 nm) in YPD medium. Total RNA was extracted according to a protocol previously described [64]. To ensure the absence of trace DNA in the samples, extracted RNA were treated with DNase and verified by conventional PCR, using primers DAN2UP and DAN2DW. cDNA were generated using an iScript cDNA synthesis Kit (Bio-Rad). Appropriately diluted cDNA from each strain were amplified using iQ SYBR Green Supermix (Bio-Rad) and a set of 13 primer pairs (Table S3) to follow the expression of 13 oxido-reductases. Expression of *ACT1*, *CDC11* and *LRE1* were used as internal controls. Quantitative RT-PCR reactions were carried out in the CFX96 Bio-Rad Thermal cycler. mRNA relative levels were calculated with Bio-Rad CFX Manager software. The oxido-reductase mRNA levels were normalized with respect to the *ACT1*, *CDC11* and *LRE1* mRNA levels. mRNA levels of oxido-reductase genes were calculated as ratios relative to that of a wild-type strain (set as 1).

### Trx2 polyclonal antibody

The BL21(DE3) *E. coli* strain (New England Biolabs) was transformed with plasmid p1672 (*TRX2*). Transformed cells were grown with shaking at 37°C in LB in the presence of kanamycin (30 mg/liter). When the OD (580 nm) of the culture reached 0.6, isopropyl-ß-D-thiogalactoside (IPTG) was added to a final concentration of 0.8 mM and growth was continued overnight at 18°C. Cell lysates were prepared using a French Press. Trx2-6His was purified by affinity chromatography on a Ni-column (Qiagen). Then, the 6-His tag was cleaved off with tobacco etch virus protease (TEV) and the uncleaved material was purified by passage through a Ni-column. The Trx2 lacking the His tag was used for antibody production in rabbits by a commercial facility (Agro-Bio).

### Western blotting

5 ml of cell cultures at logarithmic phase in complete medium were acid-quenched with 20% trichloroacetic acid (TCA, Sigma) at 4°C. Cells were harvested, resuspended in 1 ml of 20% TCA and pelleted by centrifugation. For protein extraction, the cell pellet was resuspended in 400 µl of 20% TCA and lysed with glass beads (200 µl) by vortexing for 5 min at 4°C. The lysed cells were centrifuged and the pellet was washed with cold acetone. The dry pellet was resuspended in 50 µl of TES buffer (100 mM Tris-Cl pH 8.8, 10 mM EDTA, 1% SDS) containing 30 mM 4-acetamido-4’-maleimidylstilbene-2,2’ -disulfonic acid, disodium salt (AMS, Molecular Probe) [38]. Following a 2 h incubation at 37°C with agitation in the dark, insoluble protein was removed by centrifugation. Each sample containing about 30 µg of protein was prepared with NuPAGE LDS sample buffer and was separated on a 12% NuPAGE Novex Bis-Tris gel with MES SDS buffer (Life Technologies). Upon transferring to a nitrocellulose membrane, Trx1 or Trx2 were probed with a rabbit anti-Trx1 antibody (a generous gift of C. Grant) or rabbit anti-Trx2 antibody, respectively and a secondary anti-rabbit IgG (IRDye, LI-COR) and then analyzed by quantitative immunoblot using an Odyssey Infrared Imaging System (LI-COR).

### Isolation of suppressors

Strains GF5297 and GF5305 (Table S1) were streaked out on YPD plates supplemented with Tween 80 and ergosterol and incubated under anaerobic conditions at 30°C. Ten single colonies from each strain were inoculated into YPD-Tween 80-ergosterol and grown anaerobically for 4 days. 100 µl of each culture were then plated onto YPD and incubated aerobically. About 3 to 40 colonies appeared on each plate after 3 days at 30°C. One colony from each plate was retained and these were named GF5297-1 to GF5297-10 and GF5305-1 to GF5305-10. To determine whether suppression was due to single mutations, the suppressor strains were crossed with strains GF5265 or GF5269 and the meiotic products of the diploids obtained were analyzed. The proportions of the three types of tetrads (tetratypes, parental ditypes and non-parental ditypes) suggested that the putative suppressor mutations were single mutations that were not linked to the *RAD51* locus. For determining whether the 20 suppressor mutations were recessive or dominant, the suppressor strains were crossed individually with strains GF5335 or GF5336. All diploids obtained were unable to grow on 5-FOA plates, suggesting that the suppressor mutations were recessive.

### Identification of the suppressor mutation of strain GF5305–6

To clone the *sup* gene from the suppressor-containing strain GF5305-6, we used the red/white colony assay described by Zhao et al [47]. We first disrupted genes *ADE2* and *ADE3* of strain GF5305-6, yielding strain GF5374. Plasmid p1591 was introduced by transformation, yielding strain GF5377. GF5377 colonies were red on SC-uracil, 8% glucose medium but became white or sectored on YPD, 8% glucose plates because plasmid p1591 (*TSA1, ADE3*) was easily lost without selection. Strain GF5377 was transformed with a CEN-*LEU2* plasmid-based genomic library (ATCC number 77162). Approximately 40,000 transformants were selected on SC-leucine, 8% glucose plates, of which 137 appeared red or pink. These 137 strains were restreaked on SC-leucine, 8% glucose plates and 21 strains continued to appear red or pink. These 21 strains were then tested for growth on SC-leucine, 5-FOA plates. Plasmids from the seven strains that failed to grow on SC-leucine, 5-FOA plates were recovered into *E. coli* using standard methods for further analysis.

### Fluorescence microscopy

Exponentially growing cells expressing Yap1-GFP under the control of native Yap1 promoter were washed with PBS, resuspended in mounting solution (75% glycerol in PBS) containing 4′,6-diamidino-2-phenylindole (DAPI) at concentration of 50 µg/ml, and mounted on a glass slide covered with polylysine (Sigma). Fluorescent images were captured with a Leica microscope (DMRXA) equipped with a cooled CCD camera MicroMAX (Princeton Instruments) under control of the MetaMorph software (Molecular Devices). Images obtained were processed using ImageJ software.

### Determination of dNTP levels

Approximately 3.7×10^8^ (as determined by the optical density at 600 nm [OD_600_]) cells were harvested by filtration through 25-mm white AAWP nitrocellulose filters (0.8 mm; Millipore AB). The filters were immersed in 700 µl of ice-cold extraction solution (12% TCA, 15 mM MgCl_2_) in Eppendorf tubes and frozen in liquid nitrogen. The following steps were carried out at 4°C. The tubes were vortexed for 30 s, incubated for 15 min, and vortexed again for 30 s. The filters were removed, and 700 µl supernatants were collected after centrifugation at 20,000×g for 1 min and added to 800 µl of ice-cold Freon-trioctylamine mixture (10 ml of 99% pure Freon [1,1,2-trichlorotrifluoroethane; Sigma-Aldrich] and 2.8 ml of 98% pure trioctylamine [Sigma-Aldrich]). The samples were vortexed and centrifuged for 1 min at 20,000×g. The aqueous phase was collected and added to 700 µl of ice-cold Freon-trioctylamine mixture. Volumes of 475 and 47.5 µl of the aqueous phase were collected. The 475-μl aliquots of the aqueous phase were pH adjusted with 1 M NH_4_HCO_3_ (pH 8.9), loaded on boronate columns (Affi-Gel 601; Bio-Rad), and eluted with 50 mM NH_4_HCO_3_, pH 8.9, 15 mM MgCl_2_ to separate dNTPs and NTPs. The eluates with purified dNTPs were adjusted to pH 3.4 with 6 M HCl, separated on a Partisphere SAX HPLC column (125 mm×4.6 mm, Hichrome), under isocratic elution with 0.35 M potassium phosphate buffer (pH 3.4; containing 2.5% [vol/vol] acetonitrile) and quantified using a LaChrom Elite HPLC system (VWR International). The 47.5-μl aliquots of the aqueous phase were adjusted to pH 3.4 and used to quantify NTPs by HPLC in the same way.

## Supporting Information

Figure S1 **Cytoplasmic and nuclear GSH/GSSG redox status measured by rxYFP.**
(TIF)

Table S1 **Yeast strains used in this study.**
(DOC)

Table S2 **Oligonucleotides used for plasmid and strain constructions.**
(DOC)

Table S3 **Primer pairs used for quantitative RT-PCR.**
(DOC)

Table S4 **Characterization of mutations affecting gene** ***TRR1*** **in the 20 suppressors.**
(DOC)

Table S5 **dNTP levels in wild-type, *trr1*Δ *trx1*Δ *trx2*Δ, *sml1*Δ *RNR1^TEF1^*, *trr1*Δ *sml1*Δ *RNR1^TEF1^* and *trx1*Δ *trx2*Δ *sml1*Δ *RNR1^TEF1^* strains.**
(DOC)

Table S6 **Average expression of** ***TSA2*** **,** ***AHP1*** **,** ***CCP1*** **and** ***YKL071w*** **in Set A, B and C.**
(DOC)

Text S1 **A tentative model.**
(DOC)

Text S2 **Plasmid constructions.**
(DOC)

Text S3 **Strain constructions.**
(DOC)
