# Supplementary figures and images for: Loss of the Thioredoxin Reductase Trr1 Suppresses the Genomic Instability of Peroxiredoxin tsa1 Mutants
Source: PLoS One. 2014 Sep 23;9(9):e108123. doi: 10.1371/journal.pone.0108123 (PMC4172583; doi:10.1371/journal.pone.0108123)

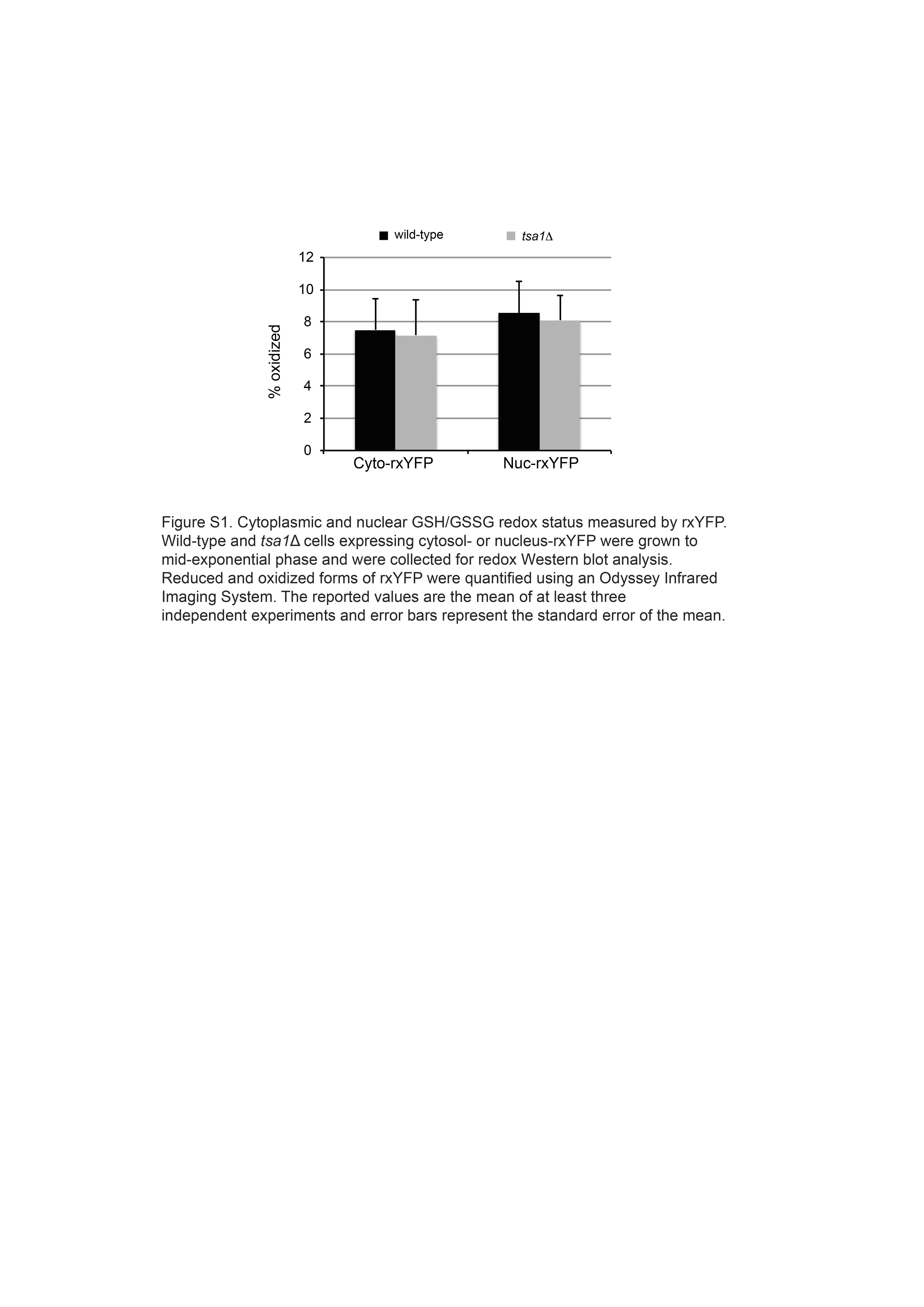

Supplement: Figure S1 — Cytoplasmic and nuclear GSH/GSSG redox status measured by rxYFP. (TIF) [file pone.0108123.s001.tif]
