# Supplementary material for: Loss of the Thioredoxin Reductase Trr1 Suppresses the Genomic Instability of Peroxiredoxin tsa1 Mutants
Source: PLoS One. 2014 Sep 23;9(9):e108123. doi: 10.1371/journal.pone.0108123 (PMC4172583; doi:10.1371/journal.pone.0108123)
Supplement: Table S1 — Yeast strains used in this study. (DOC) [file pone.0108123.s002.doc]

Table S1. Yeast strains used in this study

| Strain | Genotype |
| --- | --- |
| GF4729 | *MAT*α *ura3 leu2 trp1 lys2 HIS^+^* |
| GF4386 | *MAT*a *ura3 leu2 trp1 LYS^+^ his3* |
| GF5265 | *MAT*a *ura3 leu2 trp1 lys2 HIS^+^tsa1::TRP1* |
| GF5269 | *MAT*α *ura3 leu2 trp1 LYS^+^ his3 tsa1::TRP1* |
| GF5270 | *MAT*a *ura3 leu2 trp1 LYS^+^ his3 tsa1::TRP1* |
| GF5297 | *MAT*α *ura3 leu2 trp1 LYS^+^ his3 tsa1::TRP1 rad51::KAN* |
| GF5305 | *MAT*a *ura3 leu2 trp1 lys2 HIS^+^ tsa1::TRP1 rad51::KAN* |
| GF5305-6 | *MAT*a *ura3 leu2 trp1 lys2 HIS^+^ tsa1::TRP1 rad51::KAN sup1* |
| GF5335 | *MAT*a *ura3 leu2 trp1 lys2 HIS^+^ tsa1::TRP1 rad51::KAN* p1584 |
| GF5336 | *MAT*α *ura3 leu2 trp1 LYS^+^ his3 tsa1::TRP1 rad51::KAN* p1584 |
| GF5372 | *MAT*a *ura3 leu2 trp1 lys2 HIS^+^ tsa1::TRP1 rad51::KAN sup1 ade2*Δ |
| GF5374 | *MAT*a *ura3 leu2 trp1 lys2 HIS^+^ tsa1::TRP1 rad51::KAN sup1 ade2*Δ *ade3*Δ |
| GF5377 | *MAT*a *ura3 leu2 trp1 lys2 HIS^+^ tsa1::TRP1 rad51::KAN sup1 ade2*Δ *ade3*Δ* *p1591 |
| GF5381 | *MAT*α *ura3 leu2 trp1 lys2 HIS^+^ tsa1::TRP1 yap1::URA3* |
| GF5384 | *MAT*α *ura3 leu2 trp1 lys2 HIS^+^ yap1::URA3* |
| GF5494 | *MAT*a *ura3 leu2 trp1 LYS^+^ his3 tsa1::TRP1 rad51::KAN trr1::HIS3* |
| GF5499 | *MAT*α *ura3 leu2 trp1 LYS^+^ his3 tsa1::TRP1 trr1::HIS3* |
| GF5501 | *MAT*α *ura3 leu2 trp1 lys2 his3 tsa1::TRP1 trr1::HIS3* |
| GF5505 | *MAT*α *ura3 leu2 trp1 lys2 his3 trr1::HIS3* |
| GF5506 | *MAT*a *ura3 leu2 trp1 LYS^+^ his3 rad51::KAN trr1::HIS3* |
| GF5606 | *MAT? ura3 leu2 trp1 LYS?* *his3 tsa1::TRP1 YBP1^+^* |
| GF5630 | *MAT*α *ura3 leu2 trp1 LYS?* *his3 trx1::HIS3* |
| GF5643 | *MAT? ura3 leu2 trp1 LYS?* *HIS?* *trx2::URA3* |
| GF5652 | *MAT? ura3 leu2 trp1 LYS?* *his3 YBP1^+^* |
| GF5668 | *MAT? ura3 leu2 trp1 LYS?* *his3 trx1::HIS3 trx2::URA3* |
| GF5674 | *MAT? ura3 leu2 trp1 LYS?* *his3 tsa1::TRP1 trx1::HIS3 trx2::URA3* |
| GF5675 | *MAT? ura3 leu2 trp1 LYS?* *his3 rad51::KAN* |
| GF5719 | *MAT*α *ura3 leu2 trp1 LYS?* *his3 tsa1::TRP1 trx1::HIS3 trx2::URA3* |
| GF5888 | *MAT? ura3 leu2 trp1 LYS?* *his3 trx1::HIS3 trr1::KAN* |
| GF5898 | *MAT*a *ura3 leu2 trp1 LYS?* *his3 trx1::HIS3 trx2::URA3 trr1::KAN* |
| GF5899 | *MAT? ura3 leu2 trp1 LYS?* *his3 trx2::URA3 trr1::KAN* |
| GF5909 | *MAT? ura3 leu2 trp1 LYS?* *his3 tsa1::TRP1 trx1::HIS3 trr1::KAN* |
| GF5911 | *MAT? ura3 leu2 trp1 LYS?* *his3 tsa1::TRP1 trx1::HIS3 trx2::URA3 trr1::KAN* |
| GF5959 | *MAT? ura3 leu2 trp1 LYS?* *HIS? tsa1::TRP1 tsa2::HIS3 trr1::LEU2 rad51::KAN* |
| GF5965 | *MAT? ura3 leu2 trp1 LYS?* *his3 tsa1::TRP1 tsa2::HIS3* |
| GF5967 | *MAT? ura3 leu2 trp1 LYS?* *his3 tsa1::TRP1 tsa2::HIS3 trr1::LEU2* |
| GF5968 | *MAT? ura3 leu2 trp1 LYS?* *his3 tsa2::HIS3* |
| GF6020 | *MAT? ura3 leu2 trp1 LYS?* *his3 trx1::HIS3 trx2::URA3 rad51::KAN* |
| GF6054 | *MAT?* *ura3 leu2 trp1 LYS?* *his3 tsa2::HIS3 trr1::KAN* |
| GF6067 | *MAT*a *ura3 leu2 trp1 LYS?* *his3 tsa1::TRP1 tsa2::KAN trx1::HIS3 trx2::URA3* |
| GF6080 | *MAT*α *ura3 leu2 trp1 LYS?* *his3 sml1::KAN RNR1^TEF1^* |
| GF6084 | *MAT*a *ura3 leu2 trp1 LYS? his3 trr1::HIS3 sml1::KAN RNR1^TEF1^* |
| GF6129 | *MAT? ura3 leu2 trp1 LYS?* *his3 trx1::HIS3 trx2::URA3 sml1::KAN RNR1^TEF1^* |

The strains used for this study are derived from strain W303 and are all isogenic except for the indicated markers. ?, not determined.
