# Supplementary material for: Loss of the Thioredoxin Reductase Trr1 Suppresses the Genomic Instability of Peroxiredoxin tsa1 Mutants
Source: PLoS One. 2014 Sep 23;9(9):e108123. doi: 10.1371/journal.pone.0108123 (PMC4172583; doi:10.1371/journal.pone.0108123)
Supplement: Table S2 — Oligonucleotides used for plasmid and strain constructions. (DOC) [file pone.0108123.s003.doc]

**Table S2.** Oligonucleotides used for plasmid and strain constructions

| Name | Sequence |
| --- | --- |
| TEF1BAM | CGGGATCCGTAATTAAAACTTAGATTAGATTGCTATGCTTTCTTTC |
| TEF1ECO | CGGAATTCCACACACCATAGCTTCAAAATGTTTCTA |
| TSAHIN | GGATCCACGAAGCTTGAACTGAGCTAGTGTGAATAGC |
| TSAXHO | CAGGAATTCCTCGAGTTGCACAGAGAGCAAGGG |
| ADE2A | GCTCTAGAGCAGTGGGACGTATGATTGTTGAGG |
| ADE2B | CGGAATTCGTATGCCAAAGTCCTCGAC |
| ADE2C | CAATTCTCGAGACCTAACAGAAAAGTAGGTCAC |
| ADE2D | GGGGTACCCCGCAGTGGTGTCATTGCAGCCAC |
| ADE3A | GCTCTAGAGCAGTGAGACCAGGTAACGAGACG |
| ADE3B | CGGAATTCTATCCTCGTCCAAATGAGC |
| ADE3C | CAATTCTCGAGACTTCCATTTCCTATATGACG |
| ADE3D | GGGGTACCCCGCAGTGTCTTAGAACAGGCCATCG |
| TRX2NDE | GGGAATTCCATATGGTCACTCAATTAAAATCCGC |
| TRX2BAM | GAGCGCGGATCCCTATACGTTGGAAGCAATAGCTTGC |
| TSATRP | CGTTCTCAACGGGCCTTCCCCTCGTTCAATTGCTCACAACCAACCACAACTACATACACATACATACACACTGCAGGCAAGTGCACAAAC |
| TSACYH | TATAGATAGATAGGTATAAACGTAAAGAGTGAATTTTAAATAAGTAGTCATTTAGACAACTCTGCAAGCGTCCTGGTCTTTACCTCGAATAGG |
| RAD51S1 | TGTAGCGACAAAGAGCAGACGTAGTTATTTGTTAAAGGCCTACTAATTTGTTATCGTCATCCAGCTGAAGCTTCGTACGC |
| RAD51S2 | AAAAGAGGAGAATTGAAAGTAAACCTGTGTAAATAAATAGAGACAAGAGACCAAATACGCATAGGCCACTAGTGGATCTG |
| TRR1S1 | CCTACAAGTGCTATACAGCAAATAGCGAACAGTACGAAAGTAAACATCATATTATCAATACCAGCTGAAGCTTCGTACGC |
| TRR1S2 | GTTGGATAAGTATACAAAAATTTGAGTGTATCTATTTTATAATGGAAAATTCATGCATAGGCCACTAGTGGATCTG |
| TRR1KS1 | CCTACAAGTGCTATACAGCAAATAGCGAACAGTACGAAAGTAAACATCATATTATCAATACCAGCTGAAGCTTCGTACGC |
| TRR1KS2 | ATTTGTTGGATAAGTATACAAAAATTTGAGTGTATCTATTTTATAATGGAAAATTCATGCATAGGCCACTAGTGGATCTG |
| TRX1S1 | GTTTCCAAAACCCTGAAACTGCATTAGTGTAATAGAAGACTAGACACCTCGATACAAATACCAGCTGAAGCTTCGTACGC |
| TRX1S2 | GTAAACTATATATAACAAACACAGTATAGAAACACAATATATCGGTCATTGGGTGAGTGCATAGGCCACTAGTGGATCTG |
| TRX2A | GCTCTAGAGCAGTGCATAACTTGAGTGCCAGTG |
| TRX2B | CGGAATTCGATATCGTAGACTCTCGTG |
| TRX2C | CCGCTCGAGGGCGGTAAGGAGG |
| TRX2D | GGGGTACCCCGCAGTGAATACCAAGAGCATGACTGAC |
| TSA2S1 | CACTATTACTGTTTTTTGCTCAAGAATATATTAGCCTTACAAGAACGTAAAAAACCAATCCCAGCTGAAGCTTCGTACGC |
| TSA2S2 | ATATATAGGGTGATGTATTTTTAATTATTTAATAGGGCCTAGCGTTATCGTGCGAAGAGCATAGGCCACTAGTGGATCTG |
| YAP1A | GCTCTAGAGCAGTGCAAGAGGTCGCTGGATGTCG |
| YAP1B | CGGAATTCCGGCAAATTAGGATCTCGCCTTGC |
| YAP1C | CCGCTCGAGCGGCAGTAGTTTACAGAATGCTG |
| YAP1D | GGGGTACCCCGCAGTGAGGTGATACAATCTACCTAC |
| YBP1A | CAGCTCAAGTAGCTTTCTGTC |
| YBP1B | CTTTTCTATCCATGTATCTATGTCCG |
| YBP1G | CACCTTAGAGAATCTATAAACTTGGC |
| HIS S1 | GAAATTGTGGATAAAAACGAATCTCTAAATGACTATTTTACTGGTATAAAATGAAGTTCTCCAGCTGAAGCTTCGTACGC |
| HIS S2 | GCTATAAAACCAGAAACGAGTATGGCATGTTCAATATCTTGGTGGAAATGAAATAGGCATAGGCCACTAGTGGATCTG |
| SML1KS1 | GCTCCTTTGTGATCTTACGGTCTCACTAACCTCTCTTCAACTGCTCAATAATTTCCCGCTCCAGCTGAAGCTTCGTACGC |
| SML1KS2 | GAAAAGAACAGAACTAGTGGGAAATGGAAAGAGAAAAGAAAAGAGTATGAAAGGAACTGCATAGGCCACTAGTGGATCTG |
| RNRBAM | GAAGCTTCGTGGATCCATGTACGTTTATAAAAGAGACGGTCG |
| RNRNOT | TAGTTCTAGAGCGGCCGCTTAACCCGAACACATTTCACAAGCTTCTG |
| 348 | GAAAGAGGGGTTGTCATCAATGCAGAAGACGTTCAATTAGCTTTGAATAAGCATATGAACCGGATCCCCGGGTTAATTAACAGTAAAGGAG |
| 349 | TAATTGTAACATTATAGAAAAAGTTCTTTCGGTTACCCAGTTTTCCATAAAGTTCCCGCTGAATTCGAGCTCGTTTAAACTGGATGGCGGC |
