# Supplementary material for: Loss of the Thioredoxin Reductase Trr1 Suppresses the Genomic Instability of Peroxiredoxin tsa1 Mutants
Source: PLoS One. 2014 Sep 23;9(9):e108123. doi: 10.1371/journal.pone.0108123 (PMC4172583; doi:10.1371/journal.pone.0108123)
Supplement: Table S3 — Primer pairs used for quantitative RT-PCR. (DOC) [file pone.0108123.s004.doc]

Table S3. Primer pairs used for quantitative RT-PCR

| Name | Sequence |
| --- | --- |
| DAN2UP | GAGTGAGCTCACATCTTCAC |
| DAN2DW | CAGTACGTGTCTCAACTGG |
| TSA2-1 | CAAGCCCCACCATTTAAGAA |
| TSA2-2 | GAAAAAGCCAATGGGACAAA |
| AHP1-1 | CTAACCAAGCGTGGGCTAAG |
| AHP1-2 | GACGGCTAATTCGAAACCAA |
| CTT1-1 | AAAGAGTTCCGGAGCGTGTA |
| CTT1-2 | ACGGTGGAAAAACGAACAAG |
| GRX1-1 | ATCTTCGTCGCATCCAAAAC |
| GRX1-2 | TAACGCAGCCTGAATGTCTG |
| GRX2-1 | TCTGATTGGCCAAAAGGAAG |
| GRX2-2 | TGGATTTGGGAACGTTCAAT |
| GLR1-1 | ATTTTCCCCGAAAACATTCC |
| GLR1-2 | TATAACCAGCGCCAACAACA |
| GSH1-1 | AGGCGTGGTGAAAAAGTTTG |
| GSH1-2 | TCGCCTCTTTGTCTTCTGGT |
| CCP1-1 | GCTGCTTTGGCTTCCACTAC |
| CCP1-2 | TCGTCATCTTCCCTCAGCTT |
| YKL071w-1 | TATGCGGTAAAGACGCTCAG |
| YKL071w-2 | AGCGCAGAGGCACTTTCTTC |
| TSA1-1 | TTGGAAGGCTTCAACCAATC |
| TSA1-2 | AAGAAGGTGTCGCCTTGAGA |
| TRR1-1 | ACAGGCGCTTCTGCTAAGAG |
| TRR1-2 | CAGAGTCACCACCACCAATG |
| TRX1-1 | TGCAATTGCTCAAGACAAGC |
| TRX1-2 | CATCACCCAATTCATCGACA |
| TRX2-1 | GGGCCATGTAAAATGATTGC |
| TRX2-2 | GGCATGGAAGAAACTTCAGC |
| ACT1-1 | CGGTGATTTCCTTTTGCATT |
| ACT1-2 | CTGCCGGTATTGACCAAACT |
| CDC11-1 | TCATCCCTGTGATCAGCAAA |
| CDC11-2 | GGCAAGTTCCATCTGTCGAT |
| LRE1-1 | CTGTGACTCGCAATCTCCAA |
| LRE1-2 | CTGGTGTTGCGGGAGTAGAT |
