# Supplementary material for: Loss of the Thioredoxin Reductase Trr1 Suppresses the Genomic Instability of Peroxiredoxin tsa1 Mutants
Source: PLoS One. 2014 Sep 23;9(9):e108123. doi: 10.1371/journal.pone.0108123 (PMC4172583; doi:10.1371/journal.pone.0108123)
Supplement: Table S4 — Characterization of mutations affecting gene TRR1 in the 20 suppressors. (DOC) [file pone.0108123.s005.doc]

|  |
| --- |
| |  | | --- | | Table S4. Characterization of mutations affecting gene *TRR1* in the 20 suppressors | | | Suppressor | Codon affected | Resulting codon | | --- | --- | --- | | *sup1* | ARG70 | Stop | | *sup2* | GLU66 | Stop | | *sup3* (=*sup16*) | GLY287 | SER | | *sup4* | TYR209 | Stop | | *sup5* | LEU222 | Stop | | *sup6* | GLN74 | PRO | | *sup7* (=*sup20*) | ALA308 | PRO | | *sup8* (=*sup15*) | ALA118 | PRO | | *sup9* | GLY304 | SER | | *sup10* | GLU73 | Stop | | *sup11* | LYS197 | Stop | | *sup12* | CYS167 | Stop | | *sup13* | LEU173 | TRP | | *sup14* | LYS179 | Stop | | *sup15* (=*sup8*) | ALA118 | PRO | | *sup16* (=*sup3*) | GLY287 | SER | | *sup17* | GLY302 | ASP | | *sup18* | TYR209 | Stop | | *sup19* | TYR314 | Stop | | *Sup20* (=*sup7*) | ALA308 | PRO | | |  | |
|  |
|  |
|  |
|  |
|  |
|  |
