# Supplementary material for: Loss of the Thioredoxin Reductase Trr1 Suppresses the Genomic Instability of Peroxiredoxin tsa1 Mutants
Source: PLoS One. 2014 Sep 23;9(9):e108123. doi: 10.1371/journal.pone.0108123 (PMC4172583; doi:10.1371/journal.pone.0108123)
Supplement: Table S5 — dNTP levels in wild-type, trr1Δ trx1Δ trx2Δ, sml1Δ RNR1TEF1, trr1Δ sml1Δ RNR1TEF1 and trx1Δ trx2Δ sml1Δ RNR1TEF1 strains. (DOC) [file pone.0108123.s006.doc]

Table S5. dNTP levels in wild-type, *trr1*Δ*,* *trx1*Δ *trx2*Δ, *sml1*Δ *RNR1^TEF1^*, *trr1*Δ *sml1*Δ *RNR1^TEF1^* and *trx1*Δ *trx2*Δ *sml1*Δ *RNR1^TEF1^* strains

| Strain | Genotype | dCTP | dTTP | dATP | dGTP | Average |
| --- | --- | --- | --- | --- | --- | --- |
| GF4729 | *wild-type* | 114.45 (1) | 225.79 (1) | 112.56 (1) | 67.11 (1) | 1 |
| GF5505 | *trr1*Δ | 58.98 (0.50) | 127.66 (0.56) | 43.32 (0.38) | 40.39 (0.59) | 0.50 |
| GF5668 | *trx1*Δ *trx2*Δ | 44.95 (0.38) | 126.72 (0.56) | 59.43 (0.52) | 45.76 (0.67) | 0.52 |
| GF6080 | *sml1*Δ *RNR1^TEF1^* | 604.38 (5.29) | 1048.07 (4.65) | 654.50 (5.83) | 346.46 (5.16) | 5.22 |
| GF6084 | *trr1*Δ *sml1*Δ *RNR1^TEF1^* | 605.88 (5.30) | 994.30 (4.41) | 730.46 (6.51) | 398.15 (5.94) | 5.52 |
| GF6129 | *trx1*Δ *trx2*Δ *sml1Δ RNR1^TEF1^* | 345.23 (3.02) | 698.61 (3.09) | 486.82 (4.32) | 354.90 (5.28) | 3.93 |

Concentration of dCTP, dTTP, dATP and dGTP is presented as picomoles/10^8^ cells. The numbers in parentheses indicate the fold change of dCTP, dTTP, dATP and dGTP concentration relative to wild-type strain (set as 1).
