# Supplementary material for: Loss of the Thioredoxin Reductase Trr1 Suppresses the Genomic Instability of Peroxiredoxin tsa1 Mutants
Source: PLoS One. 2014 Sep 23;9(9):e108123. doi: 10.1371/journal.pone.0108123 (PMC4172583; doi:10.1371/journal.pone.0108123)
Supplement: Table S6 — Average expression of TSA2 , AHP1 , CCP1 and YKL071w in Set A, B and C. (DOC) [file pone.0108123.s007.doc]

Table S6. Average expression of *TSA2*, *AHP1*, *CCP1* and *YKL071w* in Set A, B and C.

|  | Set A | Set B | Set C | Ratio Set C/Set B |
| --- | --- | --- | --- | --- |
| *TSA2* | 1.1 | 11.8 | 969.4 | 82/1 |
| *AHP1* | 1.4 | 4.4 | 10.0 | 2/1 |
| *CCP1* | 1.1 | 7.4 | 49.5 | 7/1 |
| *YKL071w* | 0.9 | 9.5 | 150.6 | 16/1 |

Using data from Table 5, we calculated the average expression levels of genes *TSA2*, *AHP1*, *CCP1* and *YKL071w* for the strains belonging to either Set A, B or C. Strains of Set A: GF4729, GF5630, GF5643; strains of Set B: GF5668, GF5719, GF5898, GF5911; strains of Set C: GF5505, GF5888, GF5899, GF5909, GF5501.
