# Supplementary material for: Loss of the Thioredoxin Reductase Trr1 Suppresses the Genomic Instability of Peroxiredoxin tsa1 Mutants
Source: PLoS One. 2014 Sep 23;9(9):e108123. doi: 10.1371/journal.pone.0108123 (PMC4172583; doi:10.1371/journal.pone.0108123)
Supplement: Text S1 — A tentative model. (DOC) [file pone.0108123.s008.doc]

**Text S1. A tentative model**

We assume that the Can^R^ mutation rate can be expressed as the sum of at least three terms (Equation E): Can^R^ = a + P [dNTP] + k [DNA lesions] [dNTP], where

► a: is the fraction of Can^R^ mutations due to spontaneous alterations in DNA base chemistry [1] (mutations generated by TransLesion Polymerases are not considered in this term). These lesions are mainly repaired by the base excision repair and nucleotide excision repair pathways [2]. Since in these processes replicative DNA polymerases synthesize very short gaps of DNA, we postulate that variations of dNTP concentration will have no significant influence on the a value.

► P [dNTP]: is the fraction of Can^R^ mutations due to errors made by replicative DNA polymerases during the replication of the genome that escape mismatch repair [3]. dNTP concentration influences replicative errors, possibly in a linear manner.

► k [DNA lesions] [dNTP]: is the fraction of Can^R^ mutations generated by translesion polymerases and possibly by replicative polymerases bypassing DNA lesions [4,5].

P and k are constants that are supposed to be the same for the *trr1*Δ and *trx1*Δ *trx2*Δ contexts.

Setting the dNTP concentration in wild-type cells as 1, this one is 0.50 for *trr1*Δ, 5.52 for *trr1*Δ *sml1*Δ *RNR1^TEF1^*, 0.52 for *trx1*Δ *trx2*Δ* *and 3.93 for *trx1*Δ *trx2*Δ *sml1*Δ *RNR1^TEF1^* cells (Supplemental Table S5).

Considering the Can^R^ mutation rates (× 10^-7^) presented in Table 8, the following equations can be written for *trr1*Δ and *trr1*Δ *sml1*Δ *RNR1^TEF1^* cells:

(1) 0.84 = a + P [0.50] + k [lesions_t_] [0.50]

(2) 2.35 = a + P [5.52] + k [lesions_t_] [5.52], where [lesions_t_] is the level of DNA lesions present in *trr1*Δ and *trr1*Δ *sml1*Δ *RNR1^TEF1^* cells.

By multiplying (1) by 11.04, it comes (1’)

(1’) 9.27 = 11.04 a + P [5.52] + k [lesions_t_] [5.52]

(1’) – (2) gives 9.27 – 2.35 = 10.04 a

Thus, a = 6.92/10.04 = 0.69

For *trx1*Δ *trx2*Δ* *and *trx1*Δ *trx2*Δ *sml1*Δ *RNR1^TEF1^* cells

(3) 2.11 = a + P [0.52] + k [lesions_x_] [0.52]

(4) 11.53 = a + P [3.93] + k [lesions_x_] [3.93], where [lesions_x_] is the level of DNA lesions present in *trx1*Δ *trx2*Δ* *and *trx1*Δ *trx2*Δ *sml1*Δ *RNR1^TEF1^* cells.

By multiplying (3) by 7.56, it comes (3’)

(3’) 15.95 = 7.56 a + P [3.93] + k [lesions_x_] [3.93]

(3’) – (4) = 15.95 – 11.53 = 6.56 a

a = 4.42/6.56 = 0.67

Thus, the average value of a is: a_m_ = (0.69 + 0.67)/2 = 0.68

From (1) 0.84 = 0.68 + P [0.50] + k [lesions_t_] [0.50],

we get (11) 0.16 = P [0.50] + k [lesions_t_] [0.50]

From (3) 2.11 = 0.68 + P [0.52] + k [lesions_x_] [0.52],

we get (33) 1.43 = P [0.52] + k [lesions_x_][0.52]

By multiplying (11) by 2 and (33) by 1.92, it comes:

(7) 0.32 = P + k [lesions_t_], equivalent to 0.32 – P = k [lesions_t_]

(8) 2.74 = P + k [lesions_x_], equivalent to 2.74 – P = k [lesions_x_]

Equation (7) indicates that P may vary from 0 to 0.32.

From (7) and (8), we evaluate the variations of [lesion_x_]/[lesion_t_] = (2.74 – P)/( 0.32 – P) as a function of P (supposing that the constants k have the same value for *trr1*Δ and *trx1*Δ *trx2*Δ strains):

P = 0 2.74/0.32 = 8.56

P = 0.05 2.69/0.27 = 9.96

P = 0.10 2.64/0.22 = 12.00

P = 0.15 2.59/0.17 = 15.23

P = 0.20 2.54/0.12 = 21.16

P = 0.25 2.49/0.07 = 35.57

P = 0.30 2.44/0.02 = 122.00

P = 0.32 2.42/0.00 → ∞

The Can^R^ mutation rate of wild-type strain varies little with the variation of dNTP concentration (Table 8), we postulate that the Can^R^ mutation rate for the wild type strain follows equation E’:

Can^R^ = a + P [dNTP] + k’ [lesions_w_]

From there we get the following equations :

(5) 4.20 = a + P [1] + k’ [lesions_w_]

(6) 5.04 = a + P [5.22] + k’ [lesions_w_]

5.04 – 4.20 = 0.84 = P [4.22], then P = 0.2

If a = 0.68, from (5) it comes: k’ [lesions_w_] = 3.32, this suggests that translesion polymerases generate 3.32/4.20 = 79% of the Can^R^ mutations in wild-type cells. Indeed, it was previously observed that more than 60% of spontaneous mutations are caused by translesion synthesis [6-9].

Summarizing the findings of the model, with P = 0.20, it comes that:

for *trr1*Δ strain: (a) 0.84 = 0.68 + 0.20 [0.50] + 0.12 [0.50]

(b)* *1.00 = 0.68 + 0.20 [1.0] + 0.12 [1.0]*

(c) 2.44 = 0.68 + 0.20 [5.52] + 0.12 [5.52]

for *trx1*Δ *trx2*Δ strain: (d) 2.11 = 0.68 + 0.20 [0.52] + 2.54 [0.52]

(e)* *3.42 = 0.68 + 0.20 [1.0] + 2.54 [1.0]*

(f) 11.45 = 0.68 + 0.20 [3.93] + 2.54 [3.93]

for wild-type: (g) 4.20 = 0.68 + 0.20 [1.0] + 3.32

(*: estimated)

We have suggested that the ROS level in *trr1*Δ cells is significantly reduced compared to wild type cells because Yap1 is highly activated and numerous oxido-reductases are strongly expressed. Thus, we may surmise that the decrease of DNA lesions in *trr1*Δ cells, compared to wild type cells [(equation (g) compared with equation (b)], represents essentially the decrease of DNA lesions produced by ROS, i.e. the term [DNA lesions] in equation E should comprise mainly DNA lesions produced by ROS. Accordingly, Rossman and Goncharova [10] have shown that 90% of spontaneous mutagenesis in mammalian cells can be blocked by the over-expression of metallothionein, suggesting that the ROS are the major causes of spontaneous mutagenesis. This seems also true for wild type yeast cells. It may be worth noting that 89% of base substitutions are caused by ROS in *E. coli* cells growing in the atmosphere [11].

Considering equations (g), (b) and (a) on one hand and equations (g), (e) and (d) on the other hand, it should be possible to separate the effect of ROS reduction from the effect of dNTP concentration reduction on the Can^R^ mutation rate in *trr1*Δ and *trx1*Δ *trx2*Δ strains, compared to wild-type strain. From the Table shown below, it appears that the ROS reduction has a greater impact on the Can^R^ mutation rate of *trr1*Δ than *trx1*Δ *trx2*Δ*, * consistent with the fact that Yap1 is more activated in *trr1*Δ than *trx1*Δ *trx2*Δ*.* In contrast, the dNTP reduction has a greater effect in strain *trx1*Δ *trx2*Δ*.* As more lesions occur in *trx1*Δ *trx2*Δ than in *trr1*Δ cells (third term in Equation E), changes in dNTP concentration will have more impact on Can^R^ mutation formation in *trx1*Δ *trx2*Δ than in *trr1*Δ cells.

| Strain | Genotype | Relative dNTP concentration | Can^R^ rate × 10^-7^ | Effect on Can^R^ |
| --- | --- | --- | --- | --- |
| GF4729 | *wild-type* | [1.0] | 4.20 | 3.2 (4.20 − 1.00) |
| GF5505 | *trr1*Δ | [1.0]* | 1.00* |  |
| GF5505 | *trr1*Δ | [0.50] | 0.84 | 0.16 (1.00 − 0.84) |
| GF4729 | *wild-type* | [1.0] | 4.20 | 0.78 (4.20 − 3.42) |
| GF5668 | *trx1*Δ *trx2*Δ | [1.0]* | 3.42* |  |
| GF5668 | *trx1*Δ *trx2*Δ | [0.52] | 2.11 | 1.31 (3.42 − 2.11) |

(*: estimated)

As presented in Table 8, it is striking that an increase of dNTP concentration from 1 to 5.22 in wild-type cells does not impact strongly Can^R^ mutation rate (4.20 to 5.04). How could the error-prone translesion DNA synthesis be sensitive to variations of dNTP concentration in *trr1*Δ or *trx1*Δ *trx2*Δ strains and not in wild type strain? We may suspect that in the *trr1*Δ and *trx1*Δ *trx2*Δ contexts, redox homeostasis is disturbed as observed by Trotter and Grant [12] and Dardahlon et al. [13]. More precisely, the excess of oxidized Trxs in *trr1*Δ cells or the absence of Trxs in *trx1*Δ *trx2*Δ cells might change the redox state of proteins, including DNA damage checkpoint proteins [14]. This may stimulate post-translational modifications of PCNA and/or translesion synthesis polymerases which may confer these latter enzymes more susceptibility to variation of dNTP concentrations. Interestingly, it has been shown for *Candida albicans* that inactivation of the thioredoxin gene activates the Rad53 DNA checkpoint kinase and that the *trx1*Δ* rad53*Δ double mutation is lethal [15].

**References**

1. Friedberg E WG, Siede W, Wood R, Schultz R, Ellenberg T (2006) DNA repair and mutagenesis, 2nd Ed.

2. Boiteux S, Jinks-Robertson S (2013) DNA repair mechanisms and the bypass of DNA damage in *Saccharomyces cerevisiae*. Genetics 193: 1025-1064.

3. Reha-Krantz LJ (2010) DNA polymerase proofreading: Multiple roles maintain genome stability. Biochim Biophys Acta 1804: 1049-1063.

4. Sabouri N, Viberg J, Goyal DK, Johansson E, Chabes A (2008) Evidence for lesion bypass by yeast replicative DNA polymerases during DNA damage. Nucleic Acids Res 36: 5660-5667.

5. Stone JE, Kumar D, Binz SK, Inase A, Iwai S, et al. (2011) Lesion bypass by *S. cerevisiae* Pol zeta alone. DNA Repair (Amst) 10: 826-834.

6. Kunz BA, Ramachandran K, Vonarx EJ (1998) DNA sequence analysis of spontaneous mutagenesis in *Saccharomyces cerevisiae*. Genetics 148: 1491-1505.

7. Quah SK, von Borstel RC, Hastings PJ (1980) The origin of spontaneous mutation in *Saccharomyces cerevisiae*. Genetics 96: 819-839.

8. Ragu S, Faye G, Iraqui I, Masurel-Heneman A, Kolodner RD, et al. (2007) Oxygen metabolism and reactive oxygen species cause chromosomal rearrangements and cell death. Proc Natl Acad Sci U S A 104: 9747-9752.

9. Roche H, Gietz RD, Kunz BA (1994) Specificity of the yeast *rev3* delta antimutator and REV3 dependency of the mutator resulting from a defect (rad1 delta) in nucleotide excision repair. Genetics 137: 637-646.

10. Rossman TG, Goncharova EI (1998) Spontaneous mutagenesis in mammalian cells is caused mainly by oxidative events and can be blocked by antioxidants and metallothionein. Mutat Res 402: 103-110.

11. Sakai A, Nakanishi M, Yoshiyama K, Maki H (2006) Impact of reactive oxygen species on spontaneous mutagenesis in *Escherichia coli*. Genes Cells 11: 767-778.

12. Trotter EW, Grant CM (2003) Non-reciprocal regulation of the redox state of the glutathione-glutaredoxin and thioredoxin systems. EMBO Rep 4: 184-188.

13. Dardalhon M, Kumar C, Iraqui I, Vernis L, Kienda G, et al. (2012) Redox-sensitive YFP sensors monitor dynamic nuclear and cytosolic glutathione redox changes. Free Radic Biol Med 52: 2254-2265.

14. Le Moan N, Clement G, Le Maout S, Tacnet F, Toledano MB (2006) The *Saccharomyces cerevisiae* proteome of oxidized protein thiols: contrasted functions for the thioredoxin and glutathione pathways. J Biol Chem 281: 10420-10430.

15. da Silva Dantas A, Patterson MJ, Smith DA, Maccallum DM, Erwig LP, et al. (2010) Thioredoxin regulates multiple hydrogen peroxide-induced signaling pathways in *Candida albicans*. Mol Cell Biol 30: 4550-4563.
