# Supplementary material for: Loss of the Thioredoxin Reductase Trr1 Suppresses the Genomic Instability of Peroxiredoxin tsa1 Mutants
Source: PLoS One. 2014 Sep 23;9(9):e108123. doi: 10.1371/journal.pone.0108123 (PMC4172583; doi:10.1371/journal.pone.0108123)
Supplement: Text S2 — Plasmid constructions. (DOC) [file pone.0108123.s009.doc]

**Text S2. Plasmid constructions**

Oligonucleotides used for plasmid constructions are listed in Table S2.

Plasmids containing the deletion cassette *hisG-URA3-hisG* were constructed as follows. The 3.8-kb *hisG-URA3-hisG* *Bgl*II-*Bam*HI (blunt-ended) fragment from pNKY51 [1] was inserted in place of the *Hin*dIII-*Pme*I (blunt-ended) fragment of pFA6a-HIS3MX6 [2], yielding plasmid p980. The *Pvu*II-*Ecl*136II fragment containing *hisG-URA3-hisG* from p980 was ligated into *Eco*RV-digested pBluescript SK^+^ (Stratagene), yielding p1209.

pRS304-pro*TEF1*: The *TEF1* promoter region [3] was amplified by PCR using genomic DNA as template and primers TEF1ECO and TEF1BAM. The amplified fragment was cloned at *Eco*RI and *Bam*HI sites in pRS304 [4].

p1584 and p1591: A fragment containing the *TSA1* coding sequence was amplified using genomic DNA as template and primers TSAHIN and TSAXHO. This fragment was cloned into pRS416 (*URA3*/CEN) at *Hin*dIII and *Xho*I sites [4], yielding p1584. A 3680-bp *Bam*HI/*Bam*HI fragment harbouring the *ADE3* coding sequence was inserted at *Bam*HI site of p1584, yielding plasmid p1591.

p1596: Two short DNA fragments flanking the *ADE2* coding sequence were amplified by PCR using genomic DNA as template and primers ADE2A and ADE2B and primers ADE2C and ADE2D, respectively. These two fragments were cloned respectively into p1209 at *Xba*I-*Eco*RI and *Xho*I-*Kpn*I restriction sites flanking the *hisG-URA3-hisG* cassette, yielding p1596.

p1600: Two short DNA fragments flanking the *ADE3* coding sequence were amplified by PCR using genomic DNA as template and primers ADE3A and ADE3B and primers ADE3C and ADE3D, respectively. These two fragments were cloned respectively into p1209 at *Xba*I-*Eco*RI and *Xho*I-*Kpn*I restriction sites flanking the *hisG-URA3-hisG* cassette, yielding p1600.

p1672: The *TRX2* coding sequence was amplified using gnomic DNA as template and primers TRX2NDE and TRX2BAM. The resulting fragment was cloned into vector pSKB3 at *Nde*I and *Bam*HI sites [5], yielding p1672.

**References**

1. Alani E, Cao L, Kleckner N (1987) A method for gene disruption that allows repeated use of URA3 selection in the construction of multiply disrupted yeast strains. Genetics 116: 541-545.

2. Longtine MS, McKenzie A, 3rd, Demarini DJ, Shah NG, Wach A, et al. (1998) Additional modules for versatile and economical PCR-based gene deletion and modification in *Saccharomyces cerevisiae*. Yeast 14: 953-961.

3. Mumberg D MR, Funk M (1995) Yeast vectors for the controlled expression of heterologous proteins in different genetic backgrounds. Gene 156: 119-122.

4. Sikorski RS, Hieter P (1989) A system of shuttle vectors and yeast host strains designed for efficient manipulation of DNA in *Saccharomyces cerevisiae*. Genetics 122: 19-27.

5. Soler N, Delagoutte E, Miron S, Facca C, Baille D, et al. (2011) Interaction between the reductase Tah18 and highly conserved Fe-S containing Dre2 C-terminus is essential for yeast viability. Mol Microbiol 82: 54-67.
