# Supplementary material for: Loss of the Thioredoxin Reductase Trr1 Suppresses the Genomic Instability of Peroxiredoxin tsa1 Mutants
Source: PLoS One. 2014 Sep 23;9(9):e108123. doi: 10.1371/journal.pone.0108123 (PMC4172583; doi:10.1371/journal.pone.0108123)
Supplement: Text S3 — Strain constructions. (DOC) [file pone.0108123.s010.doc]

**Text S3. Strain constructions**

The strains used for this study are derived from strain W303 and are all isogenic except for the indicated markers (Table S1). Gene disruptions and gene replacements were made using standard PCR-based homology directed methods [1]. Yeast cells were transformed using the lithium acetate procedure [2]. Multiple combinations of mutations were usually constructed by crossing of two haploids or modifying a target gene in a diploid strain, followed by tetrad dissection. Oligonucleotides used for gene disruptions are listed in Table S2.

The *TSA1* gene was disrupted with the *TRP1-CYH2^S^* disruption cassette that was generated by PCR using plasmid p443 [3] as template and primers TSATRP and TSACYH.

The *RAD51* gene was disrupted with the KanMX4 disruption cassette that was generated by PCR using plasmid pUG6 [4] as template and primers RAD51S1 and RAD51S2.

The *TRR1* gene was disrupted with the *HIS3* gene from *S. pombe* that was prepared by PCR using plasmid pFA6A-His3MX6 [5] as template and primers TRR1S1 and TRR1S2. The *TRR1* gene was also disrupted with KanMX4 disruption cassette that was generated by PCR using plasmid pUG6 [4] as template and primers TRR1S1 and TRR1S2.

The *TRX1* gene was disrupted with the *HIS3* gene from *S. pombe* that was prepared by PCR using plasmid pFA6A-His3MX6 [5] as template and primers TRX1S1 and TRX1S2.

The *TRX2* gene was disrupted with the hisG-*URA3*-hisG disruption cassette consisting of the hisG-*URA3*-hisG flanked by DNA from upstream and downstream of the *TRX2* coding sequence. DNA from the upstream of *TRX2* was amplified using genomic DNA as template and primers TRX2A and TRX2B. DNA from the downstream of *TRX2* was amplified using genomic DNA as template and primers TRX2C and TRX2D. The resulting two fragments were cloned respectively at *Xba*I–*Eco*RI and *Xho*I-*Kpn*I restriction sites in plasmid p1209, yielding p1630. Plasmid p1630 was digested with *Bts*I, and the product containing disruption cassette was used to transform a yeast strain.

The *YAP1* gene was disrupted with the hisG-*URA3*-hisG disruption cassette with the same procedure as that for the disruption of *TRX2.* DNA from the upstream of *YAP1* was amplified using genomic DNA as template and primers YAP1A and YAP1A. DNA from the downstream of *YAP1* was amplified using genomic DNA as template and primers YAP1C and YAP1D.

The *TSA2* gene was disrupted with the *HIS3* gene from *S. pombe* that was prepared by PCR using plasmid pFA6A-His3MX6 [5] as template and primers TSA2S1 and TSA2S2. The *TSA2* gene was also disrupted with KanMX4 disruption cassette that was generated by PCR using plasmid pUG6 [4] as template and primers TSA2S1 and TSA2S2.

The *SML1* gene was disrupted with the KanMX4 disruption cassette that was generated by PCR using plasmid pUG6 [4] as template and primers SML1KS1 and SML1KS2.

The *ADE2* gene in strain GF5305-6 was disrupted with the ADE2 disruption cassette prepared from plasmid p1596 following *Bts*I digestion. Transformants were selected on SC-ura, 8% glucose dropout plates. Red colonies were spread onto 5-FOA plates to select the colonies having lost the *URA3* cassette. One resulting colony, designated GF5372, was retained.

The *ADE3* gene in strain GF5372 was disrupted with ADE3 disruption cassette prepared from plasmid p1600 following *Bts*I digestion. Transformants were selected on SC-ura, 8% glucose dropout plates. White colonies were spread onto 5-FOA plates to select the colonies having lost the *URA3* cassette. One colony, designated GF5374, was retained.

The integration of the wild-type *YBP1* gene at the *YBP1* locus of our strains was done as follows. The *YBP1* coding sequence was amplified by PCR using genomic DNA of strain RDKY3615 (S288c genetic context) [7] as template and primers YBP1A and YBP1B. The absence of mutations was verified by DNA sequencing. Then, the His3MX6 cassette was amplified using plasmid pFA6a-His3MX6 as template and primers HIS S1 and HIS S2. The PCR product was inserted just downstream of the *YBP1* coding sequence in strain RDKY3615. Using the genomic DNA of a transformant and primers YBP1A and YBP1G, a PCR reaction was carried out. The product was used to transform strain GF4386.

The strain over-expressing RNR1 was constructed as follows. *RNR1* coding sequence was amplified by PCR using genomic DNA as template and primers RNRBAM and RNRNOT. The resulting DNA fragment (2670 bp) was cloned at *Bam*HI-*Not*I restriction sites in plasmid pRS304-pro*TEF1*, yielding p1664. The plasmid was then digested with *Bsu*36I that cut the corresponding restriction site located within the *TRP1* coding sequence and the product was used to transform a yeast strain.

The strain expressing Yap1-GFP was constructed as follows. GFP coding sequence was amplified by PCR using plasmid pFA6a-GFP(S65T)-kanMX6 [5] as template and primers 348 and 349. The resulting DNA fragment that allows for precise integration of the amplified cassette at the 3’ end of the *YAP1* genomic coding sequence through homologous recombination was used to transform strain GF4386. Strain expressing Yap1-GFP and its wild-type counterpart GF4386 were found to exhibit comparable resistance to H_2_O_2_.

**References**

1. Amberg DC, Burke DJ, Strathern JN (2006) PCR-Mediated Gene Disruption: One-Step Method. CSH Protoc 2006.

2. Gietz D, St Jean A, Woods RA, Schiestl RH (1992) Improved method for high efficiency transformation of intact yeast cells. Nucleic Acids Res 20: 1425.

3. Reynaud A, Facca C, Sor F, Faye G (2001) Disruption and functional analysis of six ORFs of chromosome IV: YDL103c (QRI1), YDL105w (QRI2), YDL112w (TRM3), YDL113c, YDL116w (NUP84) and YDL167c (NRP1). Yeast 18: 273-282.

4. Guldener U, Heck S, Fielder T, Beinhauer J, Hegemann JH (1996) A new efficient gene disruption cassette for repeated use in budding yeast. Nucleic Acids Res 24: 2519-2524.

5. Longtine MS, McKenzie A, 3rd, Demarini DJ, Shah NG, Wach A, et al. (1998) Additional modules for versatile and economical PCR-based gene deletion and modification in *Saccharomyces cerevisiae*. Yeast 14: 953-961.

6. Gietz RD, Sugino A (1988) New yeast-*Escherichia coli* shuttle vectors constructed with in vitro mutagenized yeast genes lacking six-base pair restriction sites. Gene 74: 527-534.

7. Ragu S, Faye G, Iraqui I, Masurel-Heneman A, Kolodner RD, et al. (2007) Oxygen metabolism and reactive oxygen species cause chromosomal rearrangements and cell death. Proc Natl Acad Sci U S A 104: 9747-9752.
